# Preliminary Study on the Clinical and Genetic Characteristics of Hereditary Spherocytosis in 15 Chinese Children

**DOI:** 10.3389/fgene.2021.652376

**Published:** 2021-03-18

**Authors:** Chongjun Wu, Ting Xiong, Zhongjin Xu, Chunlei Zhan, Feng Chen, Yao Ye, Hong Wang, Yu Yang

**Affiliations:** ^1^The Affiliated Children’s Hospital of Nanchang University, Nanchang, China; ^2^Department of Hematology, Jiangxi Provincial Children’s Hospital, Nanchang, China; ^3^Department of Endocrine Genetics and Metabolism, Jiangxi Provincial Children’s Hospital, Nanchang, China; ^4^Department of Gastroenterology, Jiangxi Provincial Children’s Hospital, Nanchang, China

**Keywords:** hereditary spherocytosis, ANK1, SPTB, children, mutation

## Abstract

**Objective:**

To investigate the clinical and genetic characteristics of hereditary spherocythemia (HS) in Chinese children, and to analyze the potential genotypic/phenotypic associations.

**Methods:**

The clinical data and gene test results of children with HS were collected. All patients were diagnosed by gene test results, and the laboratory results were obtained before splenectomy. The data of red blood cell (RBC), hemoglobin (HB), mean red blood cell volume (MCV), mean red blood cell hemoglobin (MCH), mean red blood cell hemoglobin concentration (MCHC), and hematocrit (HCT) were statistically analyzed according to different mutation genes. Statistical methods for comparison between groups Mann–Whitney test analysis, two-terminal *p* < 0.05 was considered significant difference.

**Results:**

A total of 15 children were enrolled in our hospital, and 14 variants were found (nine variants have not been reported before), including 10 ANK1 mutations (seven ANK1 truncated mutations) and five SPTB mutations. Patients with ANK1 mutations had more severe anemia than those with SPTB mutations (significantly lower RBC, HB, MCHC, and HCT).

**Conclusion:**

This is one of the few studies on the genetic and clinical characteristics of children with HS in China. This study identified the unique genetic and clinical characteristics of Chinese children with HS and analyzed the pathogenic genotype–phenotypic association. The results confirmed that the anemia degree of HS patients caused by ANK1 was more serious than that of patients with SPTB deficiency. However, further study of the correlation between genotype and phenotype requires a larger sample size.

## Introduction

Hereditary spherocytosis (HS) is a genetic disease and the most common cause of congenital hemolytic anemia (HA) (Perrotta et al., 2008). The clinical manifestations of HS vary greatly, ranging from asymptomatic to severe hemolysis. The typical clinical manifestations are similar to other hemolytic anemias, characterized by anemia, jaundice, and splenomegaly(Hassoun and Palek, 1996). With the widespread use of genetic diagnostic techniques, many new mutations have been discovered in genes associated with HS, including ANK1, SPTB, SLC4A1, SPTA1, and EPB42 (Eber et al., 1996). These five HS related genes involved in the interaction of double red cell membrane and lipids, its coding respectively ankyrin, β/α-spectrin, band 3, and protein 4.2. Erythroid ankyrin, band 3, and protein 4.2 are the components of “band 3 complex” involving in transmembrane protein linkage, and spectrin is a component of cell membrane skeleton (Delaunay, 2007; Da et al., 2013).

Mutations in HS-related genes are scattered and non-specific, and molecular defects are significantly heterogeneous (He et al., 2018). Autosomal dominant inheritance (AD) and autosomal recessive inheritance (AR) account for 75 and 25% of HS cases, respectively (An and Mohandas, 2008). Hereditary spherocytosis occurs all over the world, with the prevalence as high as 1/2,000 in Northern Europe and North America (Bolton-Maggs et al., 2012), and the estimated prevalence in the Chinese population is 1:100,000 (Wang et al., 2015). So far, molecular genetics studies have been conducted in different populations, including populations in the United States, Europe, Brazil, Japan, and Korea. Mutations in ANK1 (∼ 50%) are the most common cause of HS, followed by SPTB (∼ 20%), SLC4A1 (∼ 15%), EPB42 (∼ 10%), and SPTA1 (∼ 5%) mutations (Delaunay, 2002; Wang et al., 2015). However, only a few studies have been described in the Chinese population (Wang et al., 2015). In several recent studies, Qin et al. (2020) investigated 35 Chinese patients with clinical suspicion of HS, Wang et al. (2020) studied 23 patients with HS, but few studies have reported on children alone and did not exclude splenectomy as a factor. This study summarized and analyzed the clinical and genetic characteristics of 15 children with HS before spleenectomy to investigate the laboratory characteristics and clinical relevance of the mutant gene or domain.

## Materials and Methods

### Patients

The clinical data of 15 HS patients admitted to Jiangxi Provincial Children’s Hospital from December 2017 to September 2020 were collected for retrospective analysis. All the children were of Han Chinese ancestry and were not related to each other. This was the first time they had been treated in an inpatient unit for unexplained anemia. None of the 15 patients underwent splenectomy and was diagnosed by peripheral blood genetic testing.

### Genetic Analysis

After informed consent of the guardians of the patients, 4 mL of peripheral venous blood (EDTA anticoagulation) and 2 mL of parental venous blood were collected from the 15 patients. Complete exon gene sequencing and Sanger verification were performed by the genetic testing company.

### Statistical Analysis

Patients were divided into ANK1 group and SPTB group based on the mutated gene. Genotypic phenotypic analysis was performed by comparing hemoglobin (Hb), red blood cell (RBC), mean red blood cell volume (MCV), mean red blood cell hemoglobin (MCH), mean red blood cell hemoglobin concentration (MCHC), red blood cell distribution width (RDW-SD), RDW-CV, absolute value of reticulocyte (RET#), reticulocyte percentage (RET), total bilirubin (TBIL), direct bilirubin (DBIL), and indirect bilirubin (IDBL) between different groups. Mann–Whitney test was used for comparison between groups, and a double-ended *P* < 0.05 was considered a significant difference.

## Results

### Clinical Characteristics of 15 Patients With HS

The detailed clinical features of the proband are shown in Table 1. Some of these children needed to be treated with blood transfusion, and some of them were given symptomatic treatment such as antibiotic during hospitalization due to the presence of infection (Supplementary Materials 1, 2). As for whether splenectomy was needed, we planned to conduct further follow-up and make a comprehensive judgment in the later stage based on factors such as spleen size, transfusion demand and age. Unfortunately, we did not test for any hormone levels in some of the children who were likely to enter puberty. The median age at diagnosis was 3 years and 6 months, and 8 of 15 patients (53.33%) were male. Red blood cell count was 2.3 (1.51–3.61) × 10^12^/L, hemoglobin count was 67 (33–109) g/L; Platelet (PLT) count was 279 (164–545) × 10^9^/L, HCT was 20.6 (10.6–31.8)%, MCV (mean red blood cell volume) was 84.8 (70.2-95.4) fl, MCH (mean hemoglobin content) was 28.5 (26–31.9) pg, MCHC (mean hemoglobin concentration) was 325 (306–358) g/L, RDW-SD (red blood cell distribution width) was 64.5 (48.2–89.1) fl, RDW-CV was 23.3 (16.3–31) %, and the absolute value of reticulocyte was 272.2 (116.22–543.492) × 10^9^/L; the percentage of reticulocyte was 13.27 (4.93–19.48)%; total bilirubin concentration was 46.5 (13.5–201.3)μmol/L; the direct bilirubin concentration was 12.93 (4.2–28.8) μmol/L; indirect bilirubin concentration was 28.8 (3.12–184.6)μmol/L. 10 cases (66.66%) had splenomegaly or gallstones.

**TABLE 1:** Clinical and laboratory features of these included hereditary spherocytosis patients.

ID	Gender	Age	RBC	WBC	HB	PLT	HCT	MCV	MCH	MCHC	RDW-SD	RDW-CV	RET#	RET	TBIL	DBIL	IDBL	Color Doppler ultrasound results
1	Female	162 months	2.3	5.58	65	248	20.5	89.1	28.3	317	89.1	28.7	352.36	15.32	156.3	12.93	143.37	Hepatosplenomegaly, gallstone
2	Female	128 months	1.51	6.49	33	201	10.6	70.2	31.9	311	54.9	24.2	146.17	9.68	36	23.57	12.43	Large liver and spleen, cholestasis
3	Female	4 months	2.43	10.71	73	545	22.9	94.2	30	319	62	18.6	322.46	13.27	20.1	7.13	3.12	Negative
4	Female	52 months	2.16	16.47	67	184	20.6	95.4	31	325	81.8	24.5	369.58	17.11	46.5	18.85	27.65	Splenomegaly
5	Female	4 months	2.83	25.63	79	279	22.6	79.9	27.9	350	64.5	23.3	248.47	8.78	43.8	15	28.8	The spleen is slightly larger
6	Male	42 months	2.44	49.16	73	330	22	90.2	29.9	332	62	20.7	422.36	17.31	51.7	28.8	22.9	Negative
7	Male	1 month	2.26	8.73	65	453	18.7	82.7	28.8	348	48.2	16.3	132.88	5.88	111.8	15.6	96.2	Negative
8	Male	57 months	2.27	6.46	62	243	18	79.3	27.3	344	59.8	21.8	116.22	5.12	19.9	9.3	10.6	Splenomegaly
9	Female	9 months	2.69	25.77	70	351	22.8	84.8	26	307	88.7	30.6	323.876	12.04	13.5	4.2	9.3	Negative
10	Male	114 months	2.9	5	82	291	22.9	79	28.3	358	53.5	18.7	142.97	4.93	41	15	26	Splenomegaly
11	Female	124 months	2.79	8.09	82	342	23.2	83.2	29.4	353	65.6	22.9	543.492	19.48	201.3	16.7	184.6	Splenomegaly, gallstone
12	Male	2 months	1.51	6.48	43	268	13.3	88.1	28.5	323	87.5	29.8	202.45	13.41	88.8	9	79.8	Splenomegaly
13	Male	168 months	3.61	5.49	109	185	31.8	88.1	30.2	343	57.2	18.2	297.464	8.24	51.4	10.5	40.9	Gallstone
14	Male	52 months	1.53	6.54	40	164	12.6	82.4	26.1	317	80.1	31	218.79	14.3	102.2	10.9	91.3	Splenomegaly
15	Male	4 months	1.93	16.14	55	335	18	93.3	28.5	306	83.3	28.9	272.2	14.13	44.4	14.1	30.3	Negative

*WBC, white blood cells (× 10^9^/L), reference range: 3.85–10;RBC, red blood cells (× 10^12^/L), reference range: 3.1–4.5; HB, Hemoglobin (g/L), reference range: 110–149; PLT, platelet (×10^9^/L), reference range: 100–320; HCT, hematocrit (%), reference range: 35–45; MCV, mean corpuscular volume (fl), reference range: 80–98; MCH, mean hemoglobin (pg), reference range: 25–35; MCHC, mean hemoglobin concentration (g/L), reference range: 300–360; RDW-SD, red blood cell distribution width (fl) reference range: 32.3–42.4; RDW-CV (%); RET #, absolute value of reticulocyte (× 10^9^/L), reference range: 25–75; RET, reticulocyte percentage (%); TBIL, total bilirubin (μmol/L), reference range: 2–22; DBIL, direct bilirubin (μmol/L), reference range: 1–8; IDBL, indirect bilirubin (μmol/L), reference range: 1–19.*

### Mutational Spectrum of HS Patients

**FIGURE 1:**
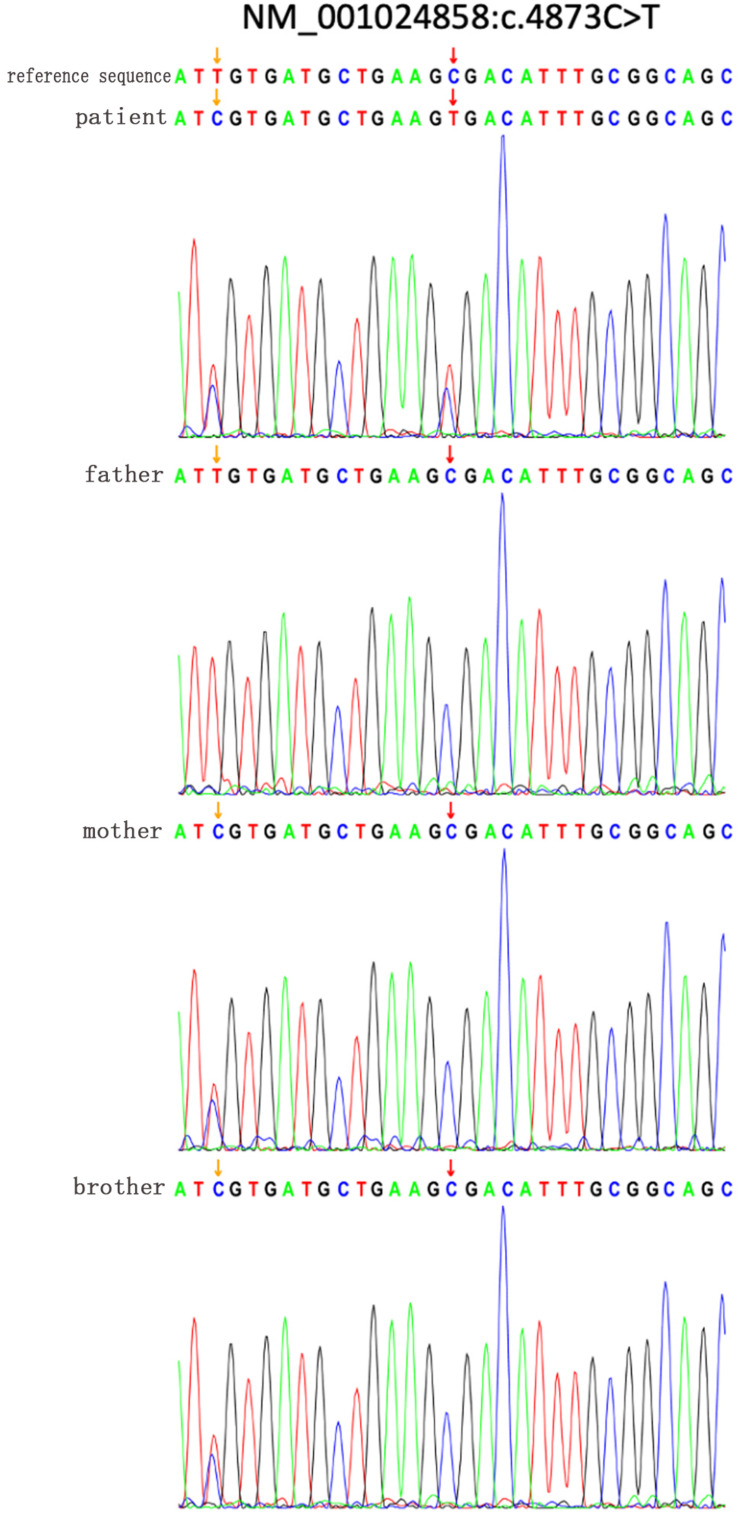
Sanger verified that the SPTB gene c.4873C > T mutation in the family of patient 1; the yellow arrow on the left indicates that the mutation c.4860T > C was inherited from a normal mother, which is of unknown significance, and its normal brother also carries it The mutation is excluded from its pathogenicity. The red arrow on the right indicates that a new heterozygous SPTB variant mutation (C. 4873C>T, 137 p.R1625X,704).

Mutation data of the 15 patients with HS were shown in Table 2. There were 10 ANK1 mutations (66.66%) and 5 SPTB mutations (33.33%), indicating that ANK1 and SPTB were the major mutated genes in Chinese children with HS. These mutations included 10 truncated mutations, three frameshift mutations, and two mutations at or near the splice site, among which the truncated mutations were dominant in the ANK1 gene. All of these variants were heterozygous, and in our study, 14 variants were found. Among them, nine variants have not been reported before, while ID1 (Agarwal et al., 2016; Shen et al., 2019), ID3 (Eber et al., 1996), ID4 (Lazzareschi et al., 2019), ID7 (Wang et al., 2017), ID12 (Wang et al., 2017), and ID14 (Aggarwal et al., 2020) have been reported in the past. The ANK1 C.4276C > T (p.r1426 ^∗^) variant was found in both patients 7 and 12. The majority of SPTB mutations (4/5) were located from introns 13 to intron 15, whereas ANK1 mutations were scattered throughout the entire gene. The core family analysis of 14 cases showed that 10 mutations were *de novo*, among which c. 2395 – 2A > G, c. 2489 _ c. 2492delTAGT, c. 2880C > A were maternal, and c. 955C > T was paternal. A four-person cohort analysis was performed in Patient 1, which detected a new heterozygous SPTB variant mutation (C. 4873C > T, p.R1625X,704), and Sanger validation was performed (Figure 1), but no family history was found. In Figure 1, the yellow arrow on the left indicates that mutation c.4860T > C is inherited from the normal mother, indicating uncertain significance, and its normal twin brother also carries this mutation, thus excluding its pathogenicity. At the same time, we further ruled out its pathogenicity through the construction of protein structure prediction model, mutation harmfulness and conservative analysis (Supplementary Materials 3–6).

**TABLE 2:** Variants detected in hereditary spherocytosispatients using next-generation sequencing.

ID	Gene	Chromosome	Nucleic acid change	Amino acid change	Proband	The father	The mother	Variation
		location	(Exon number)	(Variant Number)				type
1	SPTB	Chr14:65241215	c.4873(exon23)C > T	p.R1625X,704(p.Arg1625 Stop,704) (NM_00102 4858)	Hybrid 12/21	Wild type 0/33	Wild type 0/33 (normal)	Truncated mutant
2	ANK1	Chr8:41545696-41545697	c.4358(exon36) _ c.4359 (exon36)delAG	p.E1453 Afs*46(p.Glu1453Al afs*46) (NM_ 001142446)	Hybrid 83/170	Wild type 0/45	Wild type (normal) 0/56	Frameshift mutations
3	ANK1	Chr8:41559136-41559139	c.2489(exon22) _ c.2492 (exon22)delTAGT	p.L830Sfs*7(p.Leu830 Serfs*7) (NM_ 001142446)	Hybrid 23/47	Wild type 0/61	(patient) heterozygous 25/47	Frameshift mutations
4	ANK1	Chr8:41547849	c.4123(exon34) C > T	p.R1375X,523(p.Arg1375 Stop,523) (NM_ 001142446)	Hybrid 45/111	Wild type 0/75	Wild type (normal) 0/73	Truncated mutant
5	SPTB	Chr14:65260556	c.1825(exon13) C > T	p.Q609X,1720(p.Gln609 Stop,1720) (NM_ 00102 4858)	Hybrid 8/21	Wild type 0/19	Wild type (normal) 0/38	Truncated mutant
6	ANK1	Chr8:41559664	c.2395-2(IVS20) A > G	(NM_ 001142446)	Hybrid 22/59	Wild type 0/42	(slight) heterozygosity 31/60	Splicing site mutation
7	ANK1	Chr8:41546059	c.4276(exon35) C > T	p.R1426X,472(p.Arg1426 Stop,472) (NM_ 001142 446)	Hybrid 51/98	Wild type 0/42	Wild type 0/46	Truncated mutant
8	ANK1	Chr8:41615556	c.226(exon2) C > T	p.Q76X,1822(p.Gln76 Stop,1822) (NM_ 001142446)	Hybrid 15/34	Wild type 0/36	Wild type 0/32	Truncated mutant
9	ANK1	Chr8:41571714-41571715	c.1858(exon16) _ c.1859 (exon16)delCT	p.L620Afs*33(p.Leu620 Alafs*33) (NM_ 001142446)	Hybrid 30/86	Wild type 0/50	Wild type 0/49	Frameshift mutations
10	SPTB	Chr14:65253555	c.3128(exon15) G > A	p.W1043X,1286(p.Trp1043 Stop,1286) (NM_ 001024858)	Hybrid 87/164	Wild type 0/56	Wild type 0/50	Truncated mutant
11	SPTB	Chr14:65253803	c.2880(exon15) C > A	p.C960X,1369(p.Cys960 Stop,1369) (NM_ 001024858)	Hybrid 60/100	Wild type 0/55	hybrid 65/113	Truncated mutant
12	ANK1	Chr8:41546059	c.4276(exon35) C > T	p.R1426X,472(p.Arg1426 Stop,472) (NM_ 001142446)	Hybrid 35/69	Wild type 0/50	Wild type 0/43	Truncated mutant
13	SPTB	Chr14:65258462	c.2779(exon14) C > T	p.Q927X,1402(p.Gln927 Stop,1402) (NM_ 001024858)	Hybrid 17/43	Wild type 0/39	Wild type 0/34	Truncated mutant
14	ANK1	Chr8:41580696	c.955(exon9) C > T	p.R319X,1579(p.Arg319 Stop,1579) (NM_ 001142446)	Hybrid 22/50	hybrid 34/73	Wild type 0/41	Truncated mutant
15	ANK1	Chr8:41573376	c.1504-9(IVS13) G > A	(NM_001142446)	Hybrid 27/44	-	-	Mutations near the shear site

### Genotype–Phenotype Correlation in HS Patients

**FIGURE 2:**
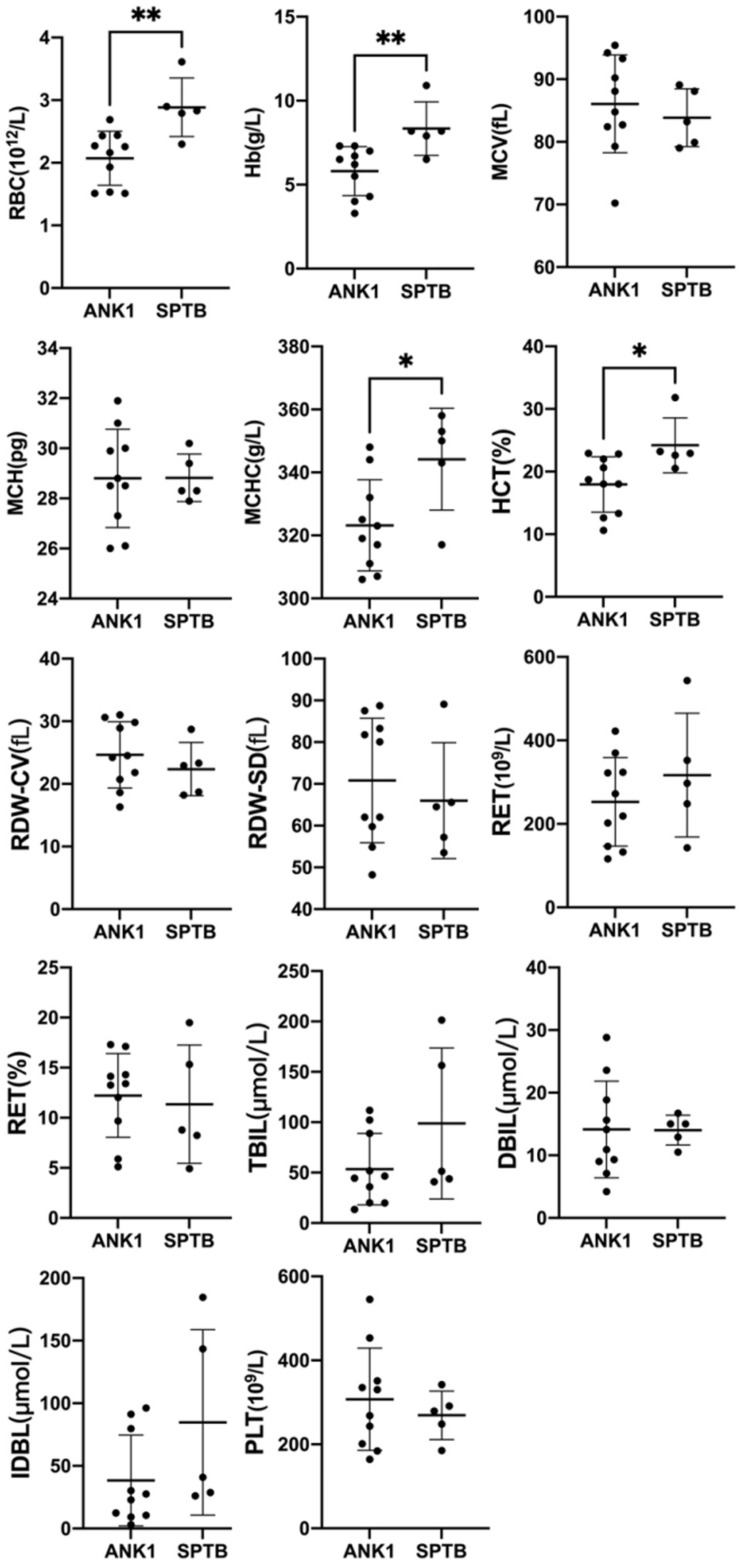
Comparison of Hb, RBC, MCV, MCH, MCHC, and other indicators between the two groups of 15 patients in our hospital. ^∗^*p* < 0.05, ^∗∗^*p* < 0.001.

A group analysis of the pathogenicity of ANK1 and SPTB gene mutations was performed on 15 patients in our hospital (Figure 2). Compared the indicators of Hb, RBC, MCV, MCH, MCHC, HCT, RDW-CV, RDW-SD, RET#, RET, TBIL, DBIL, IDBL, etc. in each group. We found that patients with ANK1 mutations had significantly lower RBC (*p* = 0.0047), HB (*p* = 0.0087), MCHC (*p* = 0.0426), and HCT (*p* = 0.0216) than those with SPTB mutations, and there was no significant difference in other indicators between the two groups (Supplementary Material 7). This suggested that patients with ANK1 mutations had more severe anemia than those with SPTB mutations. However, there were only five patients with SPTB mutation, and further study on the correlation between genotype and phenotype required a larger sample size.

## Discussion

HS is the most common non-immune hereditary hemolytic anemia with variable expression (Iolascon et al., 2019), and most HS is inherited in AD mode. The clinical presentation of HS varies widely, from asymptomatic to severe hemolysis. The typical clinical presentation is similar to other hemolytic anemia and is characterized by anemia, jaundice, and splenomegaly. Because of the significant heterogeneity of clinical manifestations of HS, it is difficult to be detected by routine laboratory examination, which is prone to misdiagnosis and missed diagnosis. No splenectomy was performed in 15 patients in this group. The median age of diagnosis was 3 years and 6 months. Hb, RBC, and HCT of all patients without splenectomy were decreased. MCH and MCHC levels were within the normal range, which was similar to the recent studies in India and Hubei (Aggarwal et al., 2020; Wang et al., 2020). The study of Michaels et al. showed that the MCHC of most HS cases was > 359 g/L (Michaels et al., 1997). However, in our study, we found that 100% of patients had MCHC < 359 g/L, similar to the study of Wang (Wang et al., 2020). In their report, it was found that 89% of patients (17/19) had MCHC < 35.9 g/dL, which further confirmed that MCHC has a poor measurement effect on HS.

HS exhibits extensive phenotype and genotype heterogeneity. The prevalence of different races and ethnic regions is different, and the molecular profiles of different regions are also different. For example, ANK1 variants account for 40–65% in the United States and Europe, and 5–10% in Japan (Perrotta et al., 2008). Here, the ANK1 variant accounted for 66.66%, which is different from that observed in the Japanese population, but similar to that in the Korean population (Park et al., 2016). In a cohort of 95 HS patients from the Netherlands, SPTA1, ANK1, and SPTB were listed as the top three genes with identified variants (van Vuren et al., 2019).

Also in China, Hubei province carried ANK1 (57%) and SPTB (43%) mutations (Wang et al., 2020). Wang et al. (2018) also found 45% ANK1 and 45% SPTB mutations. Qin et al. (2020) found ANK1 (46%) and SPTB (46%) mutations in 35 HS patients, while only 9% of patients carried SLC4A1 mutations. This implies a different geographical distribution of mutations in ANK1 and suggests that ANK1 and SPTB are the major genes in Chinese patients with HS. Since SLC4A1-HS is usually mild and often appears in adulthood, we, as a pediatric center, may have underestimated the true prevalence of SLC4A1-HS. In contrast, centers that focus primarily on adult patients may underestimate the more severe types of HS who underwent splenectomy in childhood, while adult centers never followed up.

Genotype-phenotype association analysis is rarely studied in HS patients, and it is currently controversial. Aggarwal et al. (2020); Tole et al. (2020), and others reported that the index of patients with ANK1 mutation was similar to that of SPTB group, and the type or location of the mutation in each gene could not predict the severity of the disease. Park et al. (2016) showed that SPTA1 mutations are associated with the most severe diseases, and SLC4A1 mutations are associated with the mildest diseases. At present, Qin et al. (2020) found that compared with SPTB mutation patients, the MCV and MCH levels of ANK1 mutation patients were significantly higher. In addition, the percentage of spherocytes in the peripheral blood of patients with ANK1 mutations was significantly reduced. It was found that the MCHC levels of the nonsense group, frameshift group and splicing group were significantly higher than those of the missense group, while the severity of the disease was not significantly different between the different groups. Wang et al. (2020) found no significant difference between patients with any SPTB variant. However, other groups are currently mixed with splenectomy/unresection, and the clinical manifestations of hemolytic anemia with obvious anemia can be eliminated by removing the spleen. In order to rule out splenectomy factors, none of the 15 patients in this group underwent splenectomy. The results confirmed that the degree of anemia in HS patients caused by ANK1 and SPTB defects was different. The former caused more severe anemia in HS patients and showed lower RBC, HB and MCHC levels. This is different from previous reports (Qin et al., 2020; Wang et al., 2020), but maybe the reason why splenectomies were performed more frequently in HS with ANK1 mutation (14/44) than in HS with SPTB mutation (3/31) (Park et al., 2016). However, larger sample size is needed to further investigate the association between genotype and phenotype.

ANK1 mutations are scattered throughout the entire gene. Ankyrins typically consist of three structural domains: an N-terminal domain with multiple ankyrin repeats, a central domain containing a spectrin-binding domain especially ZU5 subdomain, and a C-terminal domain with regulatory regions including death domain. It has been reported that patients with ANK1 mutations in the spectrin-binding domain has the most severe anemia compared with others (Park et al., 2016), and several loss-of-function mutations in the ZU5 subdomain have previously been reported (Kizhatil et al., 2007). Similarly, van Vuren et al. (2019) found that variants affecting spectin binding of SPTA1, ANK1, and SPTB lead to more severe phenotypes. They also found that erythrocyte deformability was correlated with HS severity. Wang et al. (2020) found that compared with other ANK1 structural domains, the variation of ANK1 death domain was correlated with low levels of MCV and MCH. However, we attempted to further investigate whether there were differences in baseline indicators such as RBC, HB, MCV, MCH, and MCHC among patients with different ANK1 domain defects, but the number of cases was too small to be statistically significant.

## Conclusion

This is one of the few studies on the genetic and clinical characteristics of children with HS in China. We found 14 mutations in SPTB and ANK1 genes in 15 Chinese children with HS. Our genotype-phenotypic association study showed that ANK1-HS was more severe than SPTB-HS in anemia. However, larger sample size is needed to further investigate the association between genotype and phenotype.

## Data Availability Statement

The original contributions presented in the study are included in the article/Supplementary Material, further inquiries can be directed to the corresponding author/s.

## Ethics Statement

The studies involving human participants were reviewed and approved by Ethics Committee of Jiangxi Provincial Children’s Hospital. Written informed consent to participate in this study was provided by the participants’ legal guardian/next of kin.

## Author Contributions

YYa: project leads and designing the project. TX, CZ, YYe, and HW: data collection. TX, CZ, YYe, and HW: data collection. ZX and FC: analysis. CW: manuscript writing. All authors contributed to the article and approved the submitted version.

## Conflict of Interest

The authors declare that the research was conducted in the absence of any commercial or financial relationships that could be construed as a potential conflict of interest.
