# Supplementary material for: Preliminary Study on the Clinical and Genetic Characteristics of Hereditary Spherocytosis in 15 Chinese Children
Source: Front Genet. 2021 Mar 18;12:652376. doi: 10.3389/fgene.2021.652376 (PMC8044778; doi:10.3389/fgene.2021.652376)
Supplement: Supplementary file 1 [file Presentation_1.pdf]

ID	clinical features	G-6-PD	Testing for Thalassemia	bone marrow puncture	Coombs testing	spherocytes	FER	Clinical treatment	(mother)Clinical symptoms/genotypes	(father)Clinical symptoms/genotypes	Inheritance
1	Anemia, jaundice, brown urine	normal	normal	unmeasured	negative	negative	124.5	Blood transfusion	Normal/ wild type	Normal/ wild type	De novo
2	Pale	-	normal	Hyperplastic anemia	negative	negative	325.80	Blood transfusion	Normal/ wild type	Normal/ wild type	De novo
3	Anemia	normal	normal	-	negative	negative	264.57	Blood transfusion	Anemia/ heterozygosity	Normal/ wild type	Mother
4	Anemia, jaundice	-	normal	Hyperplastic anemia	negative	36%	-	antibiotic, blood transfusion	Normal/ wild type	Normal/ wild type	De novo
5	Anemia	normal	normal	Hyperplastic anemia	negative	14%	401.95	Blood transfusion	Normal/ wild type	Normal/ wild type	De novo
6	Pale complexion, fever, red urine	-	normal	-	negative	negative	405.95	antibiotic	Anemia/ heterozygosity	Normal/ wild type	Mother
7	Jaundice, pale complexion	normal	normal	-	negative	negative	331.57	Blood transfusion	Normal/ wild type	Normal/ wild type	De novo
8	Fever，anemia	normal	normal	-	negative	negative	3085	antibiotic, blood transfusion	Normal/ wild type	Normal/ wild type	De novo

9	Anemia	normal	normal	Hyperplastic anemia	negative	22%	345.16	antibiotic, blood transfusion	Normal/ wild type	Normal/ wild type	De novo
10	Anemia	normal	normal	Hyperplastic anemia	negative	5%	345.38	antibiotic, blood transfusion	Normal/ wild type	Normal/ wild type	De novo
11	Jaundice, abdominal pain	normal	normal	-	negative	negative	196	antibiotic	Anemia/ heterozygosity	Normal/ wild type	Mother
12	Anemia	normal	normal	Hyperplastic anemia	negative	4%	176	Blood transfusion	Normal/ wild type	Normal/ wild type	De novo
13	Jaundice	normal	normal	-	negative	negative	52	none	Normal/ wild type	Normal/ wild type	De novo
14	pale	normal	normal	Hyperplastic anemia	negative	3%	25	Blood transfusion	Normal/ wild type	Normal/ heterozygosity	Father
15	pale	normal	normal	-	negative	negative	435	none	Anemia/ -	Normal/ -	-

Note : G-6-PD : Glucose-6-phosphate dehydrogenase; FER : Ferritin (ng/ml), reference range : 144-600 (1m) , 50-142 (6m) ,14-142 (6m-15y) , 22-322(＞15y). ID14 considers not completely explicit effects.
