# Supplementary figures and images for: Preliminary Study on the Clinical and Genetic Characteristics of Hereditary Spherocytosis in 15 Chinese Children
Source: Front Genet. 2021 Mar 18;12:652376. doi: 10.3389/fgene.2021.652376 (PMC8044778; doi:10.3389/fgene.2021.652376)

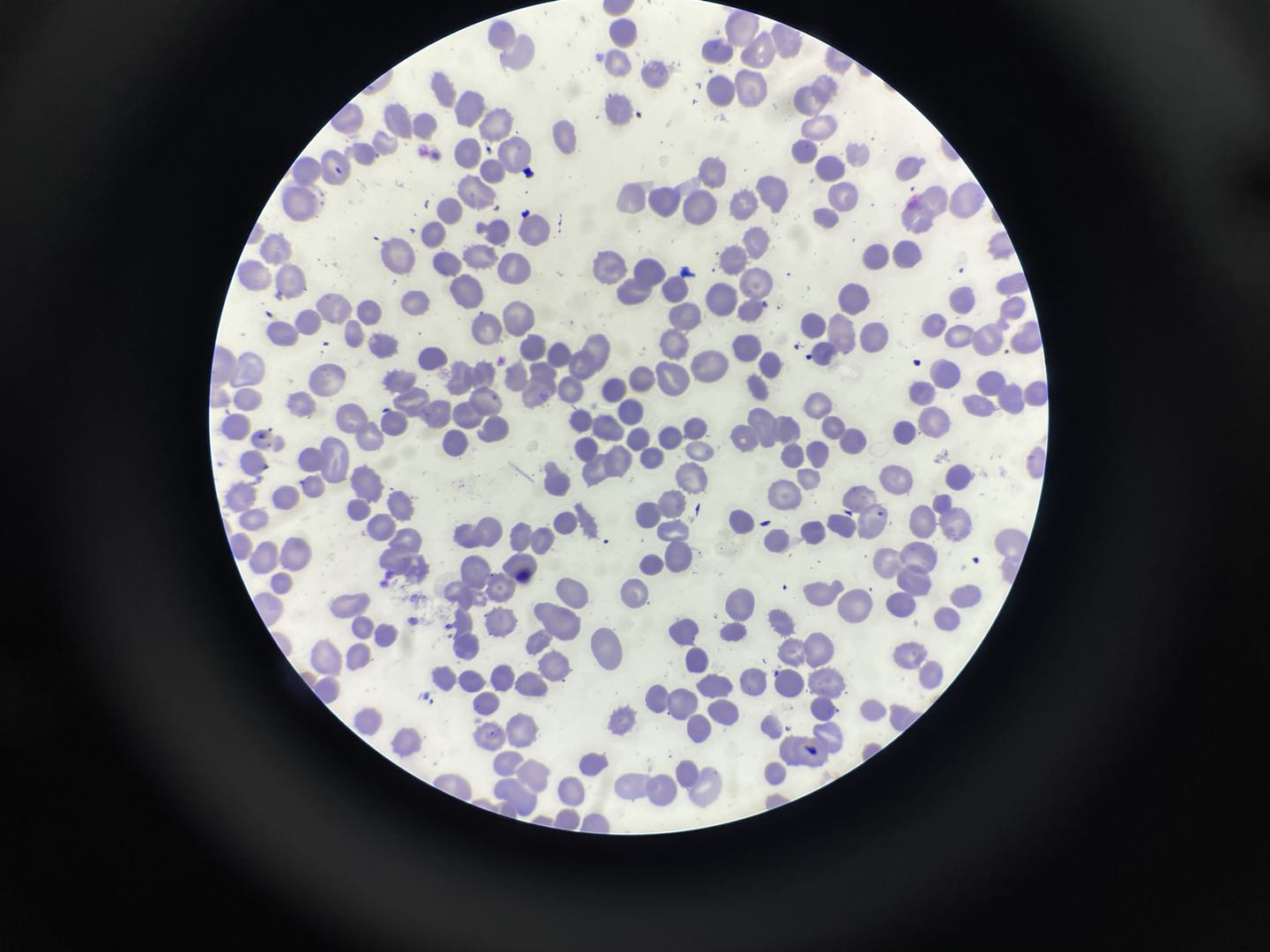

Supplement: Supplementary file 2 [file Presentation_2.zip › supplementary material 2/1-Optical microscopeú¿10í┴100 magnificationú⌐.jpg]

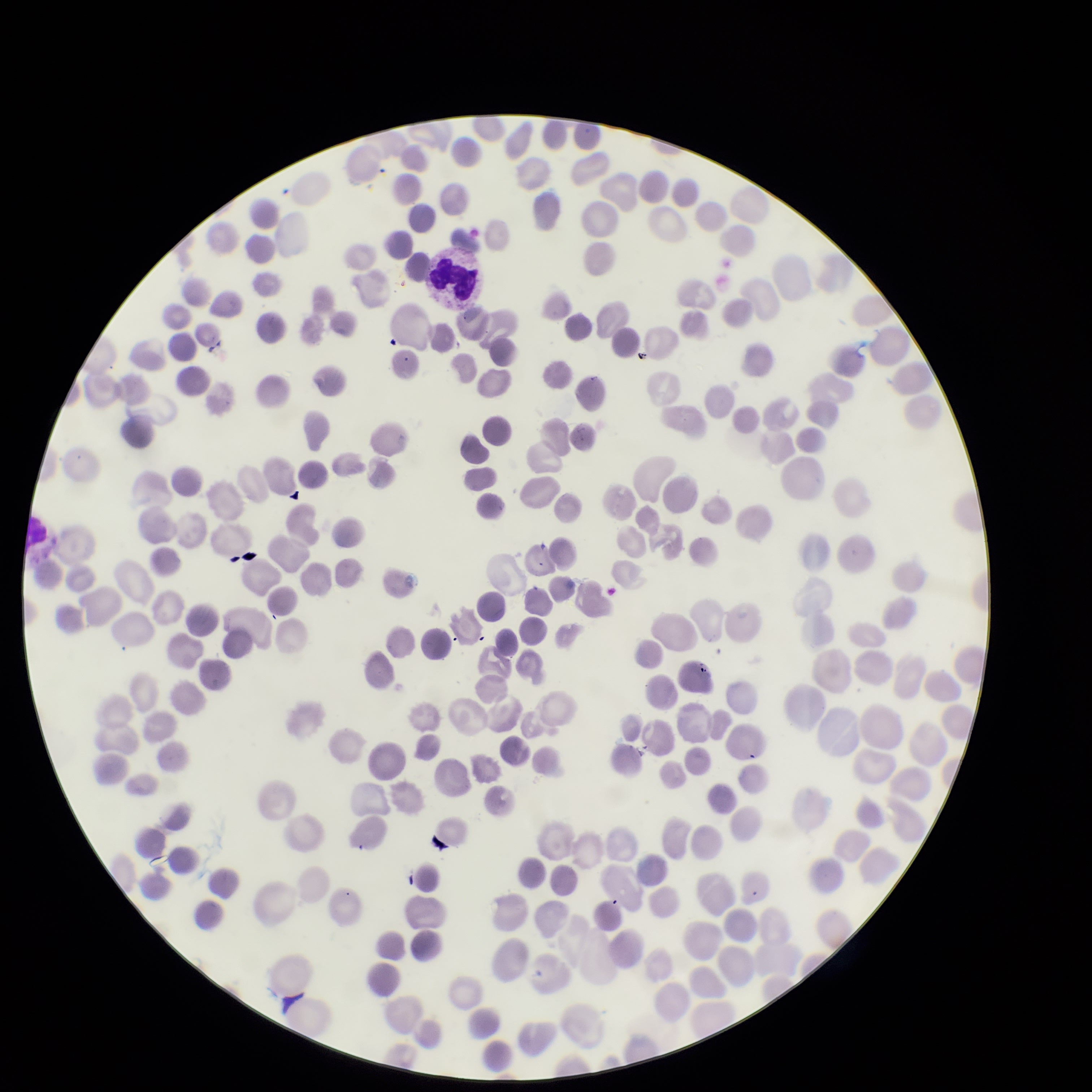

Supplement: Supplementary file 2 [file Presentation_2.zip › supplementary material 2/2-Optical microscopeú¿10í┴100 magnificationú⌐.jpg]

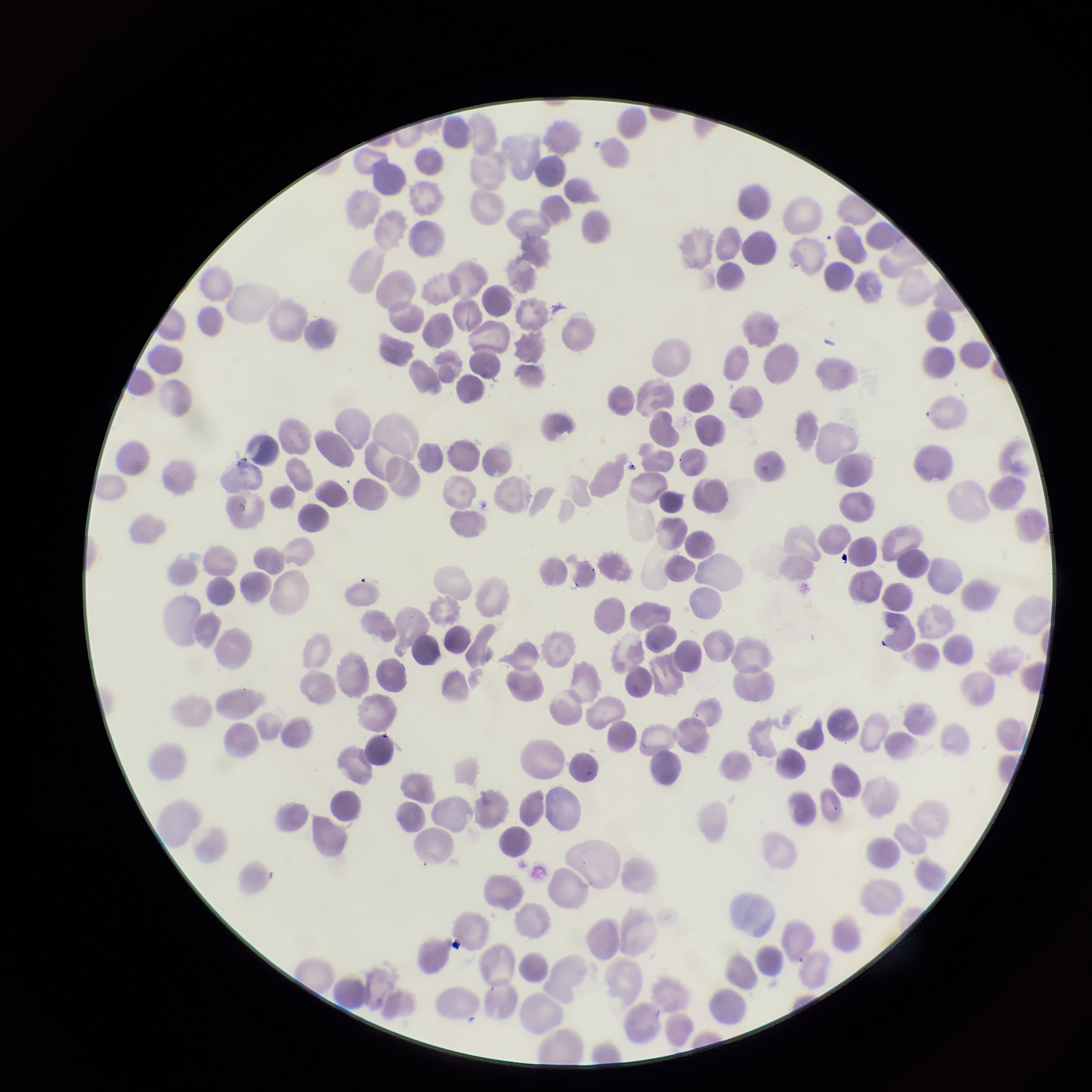

Supplement: Supplementary file 2 [file Presentation_2.zip › supplementary material 2/3-Optical microscopeú¿10í┴100 magnificationú⌐.jpg]

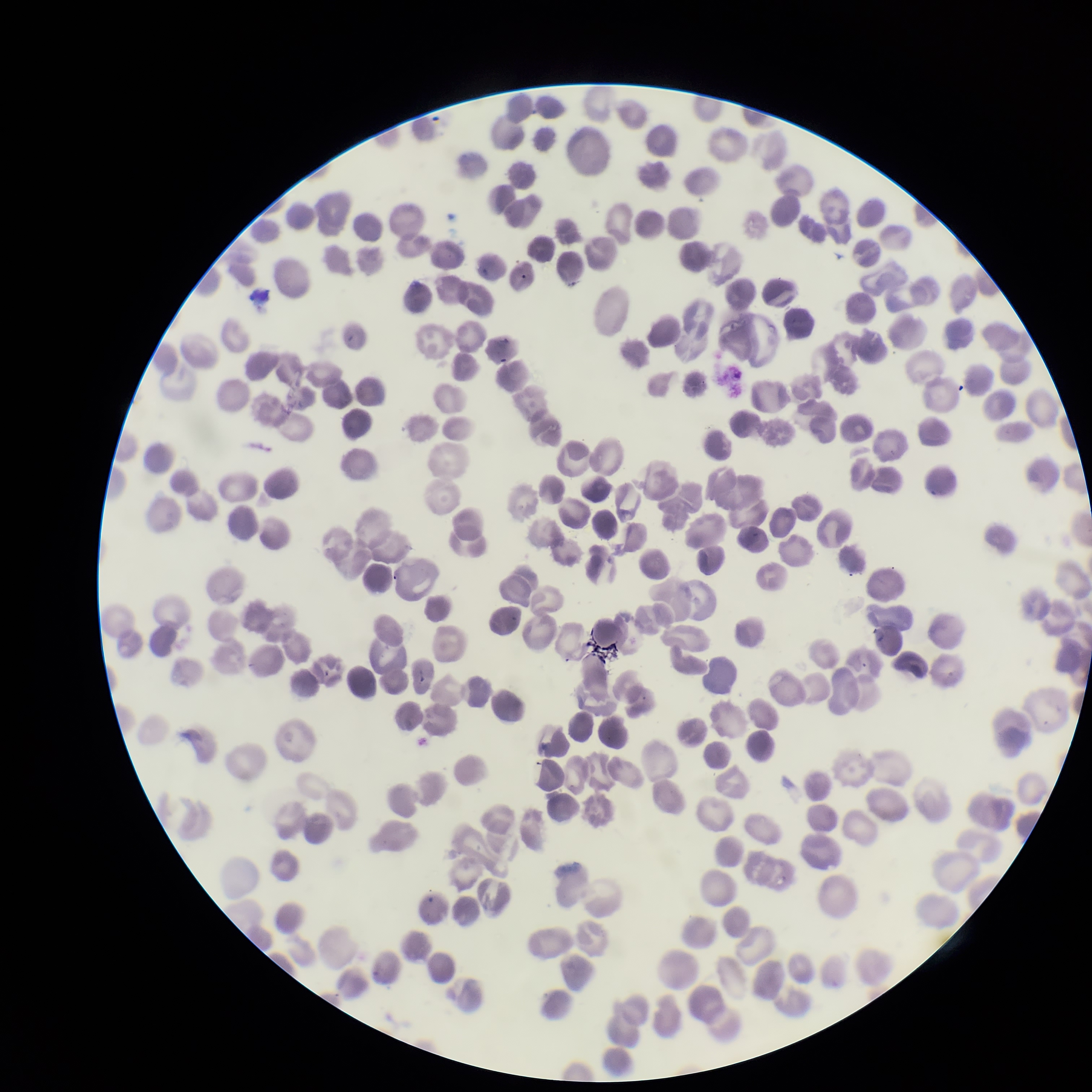

Supplement: Supplementary file 2 [file Presentation_2.zip › supplementary material 2/4-Optical microscopeú¿10í┴100 magnificationú⌐.jpg]

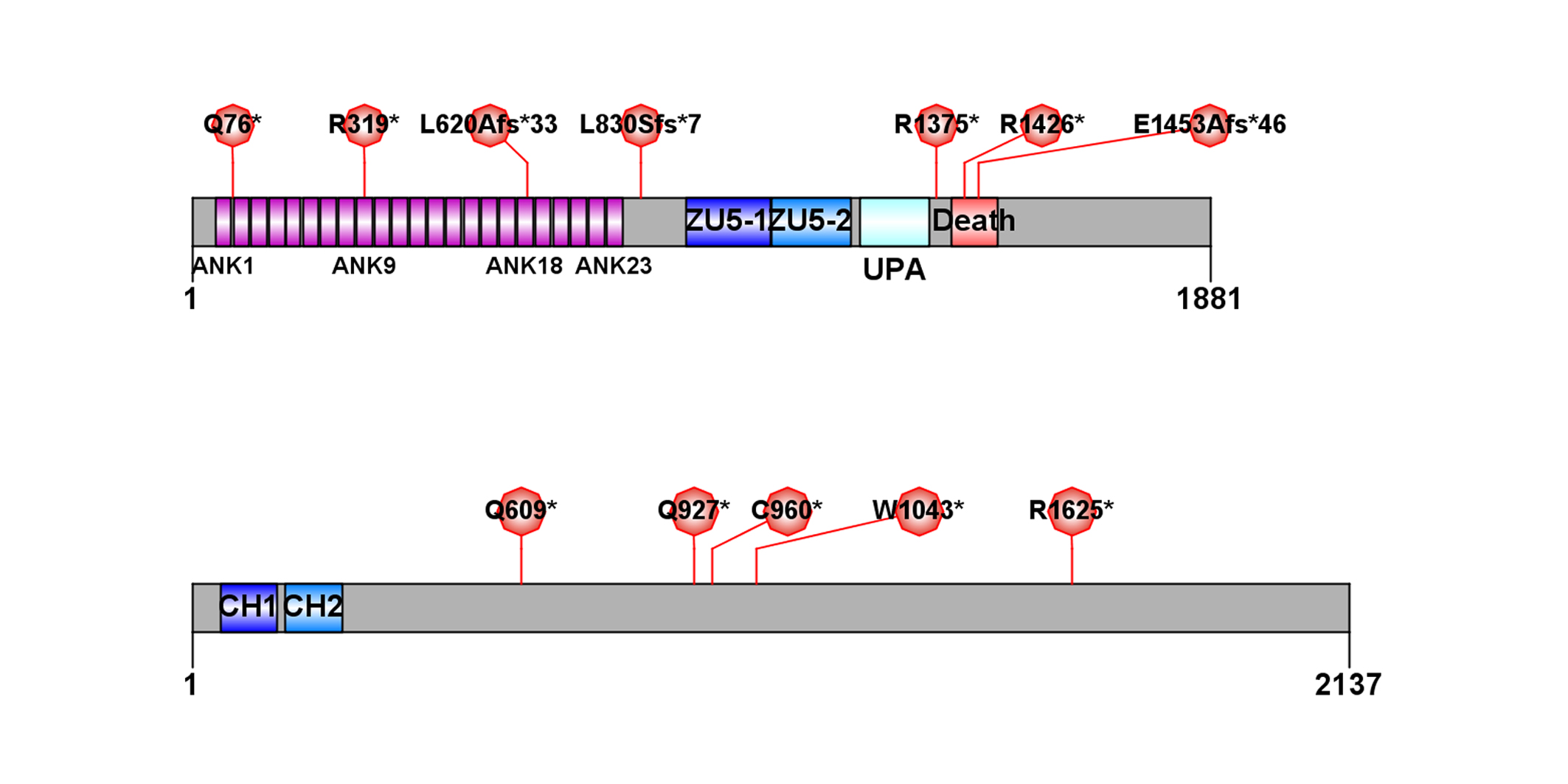

Supplement: Supplementary file 3 [file Presentation_3.zip › supplementary material 3.JPEG]

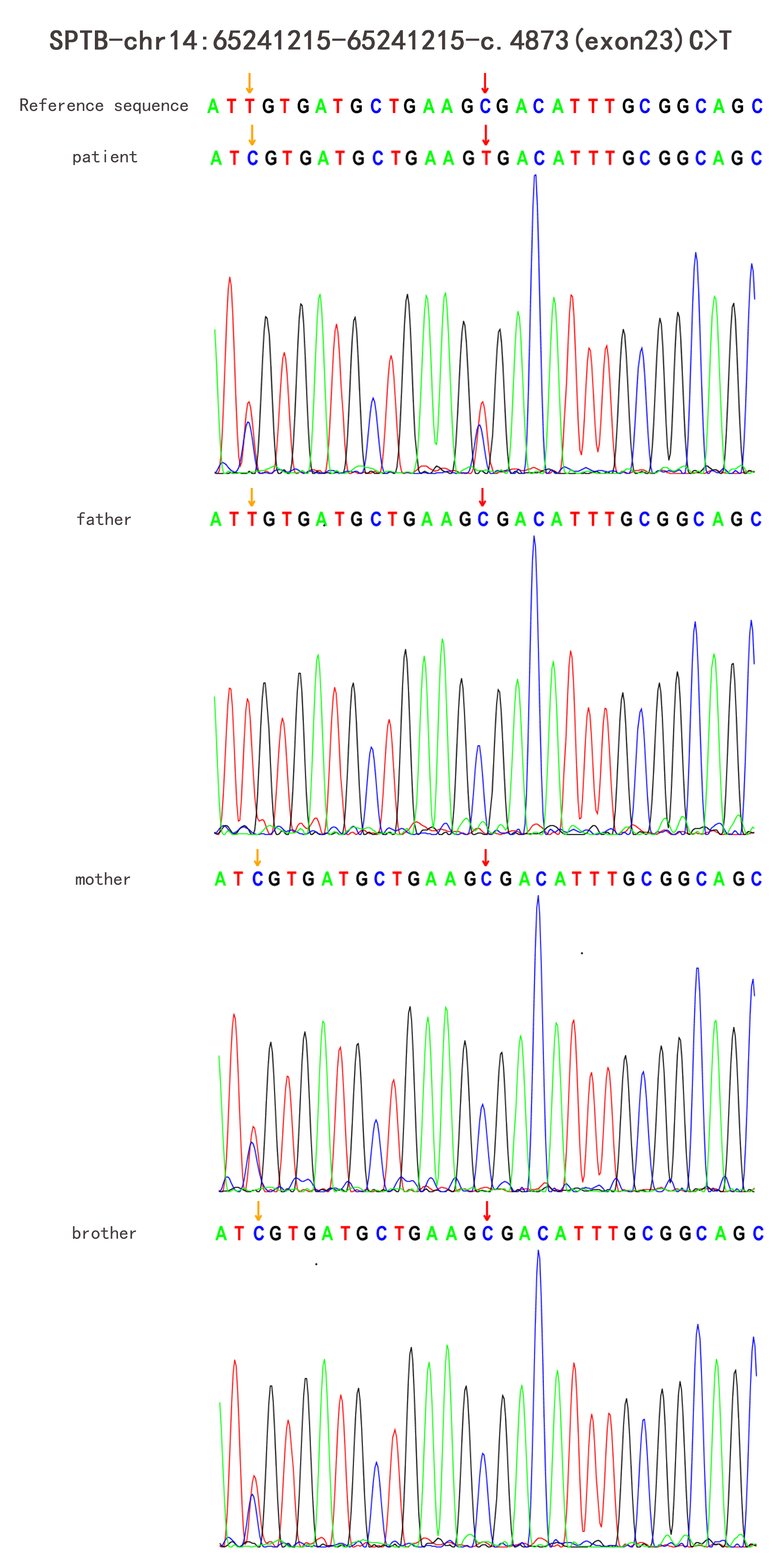

Supplement: Supplementary file 4 [file Presentation_4.zip › supplementary material 4/ID-1.jpg]

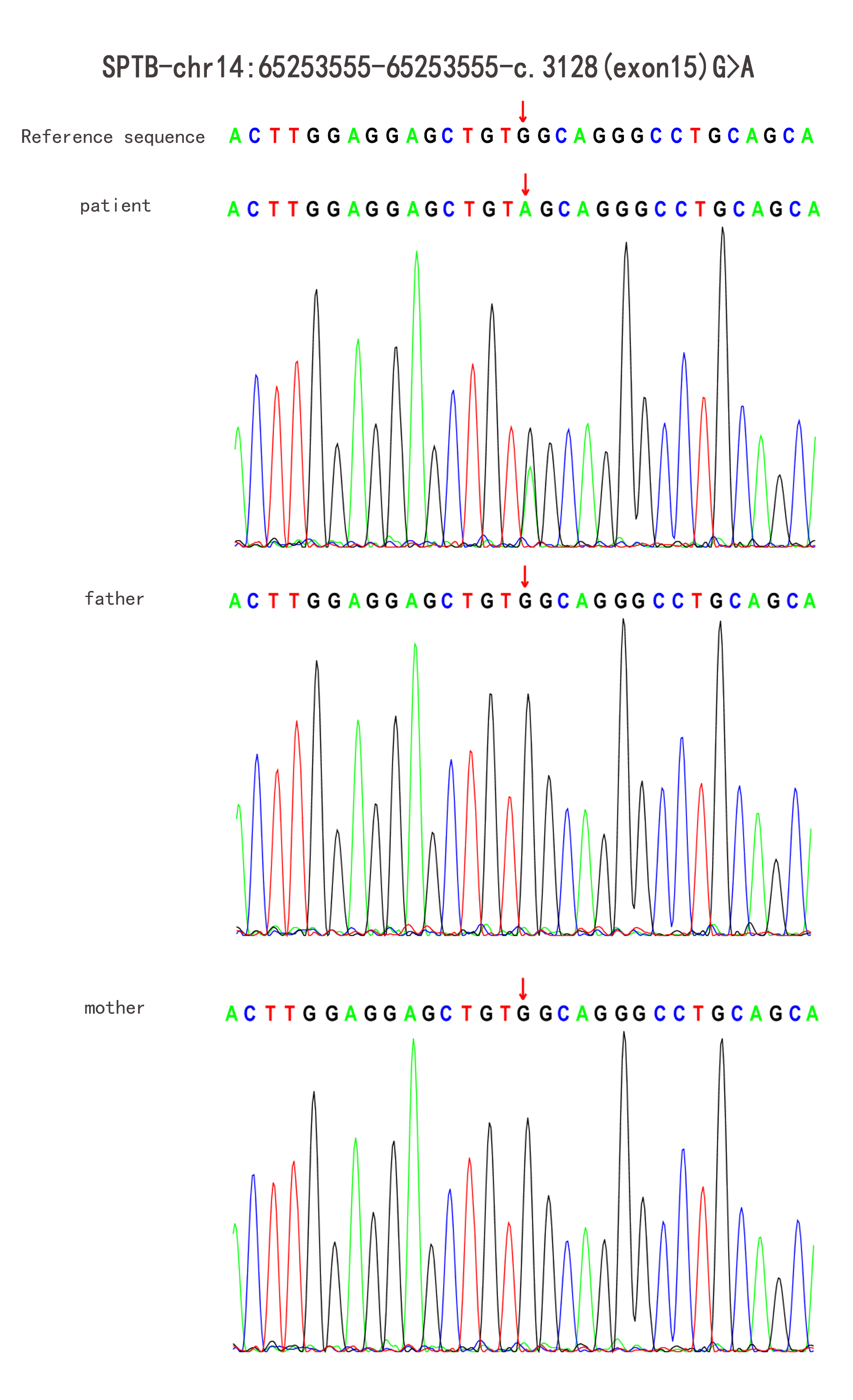

Supplement: Supplementary file 4 [file Presentation_4.zip › supplementary material 4/ID-10.jpg]

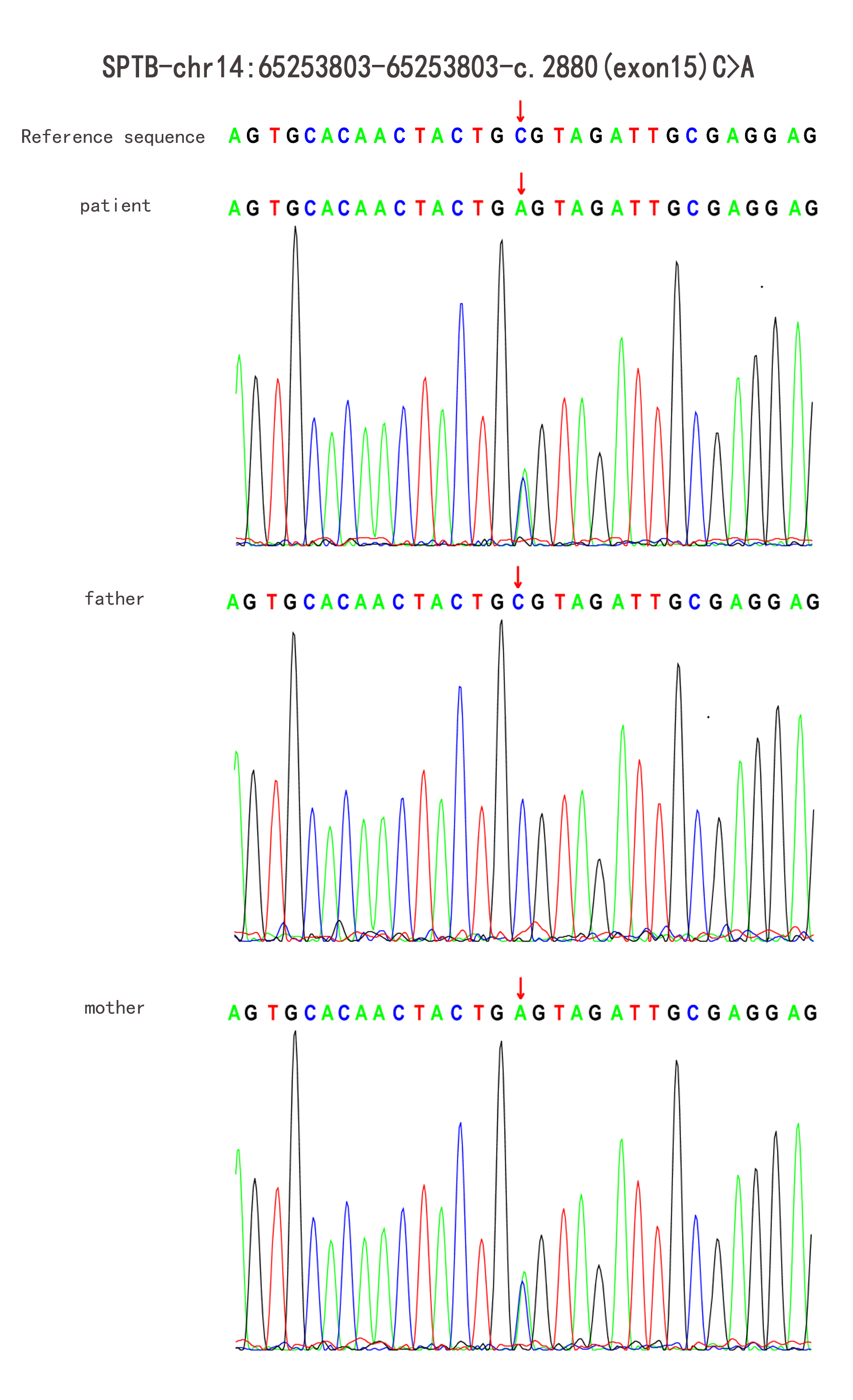

Supplement: Supplementary file 4 [file Presentation_4.zip › supplementary material 4/ID-11.jpg]

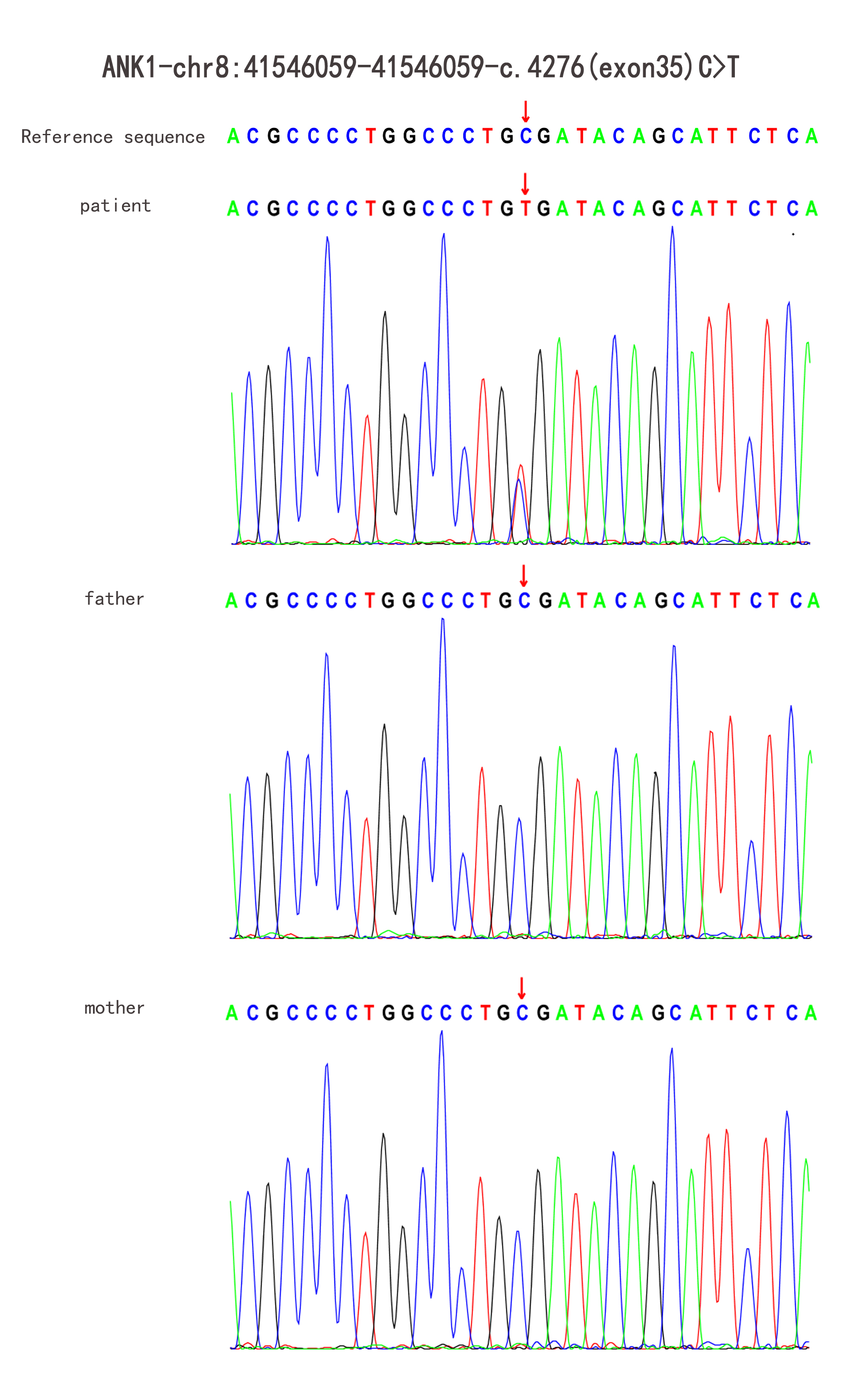

Supplement: Supplementary file 4 [file Presentation_4.zip › supplementary material 4/ID-12.jpg]

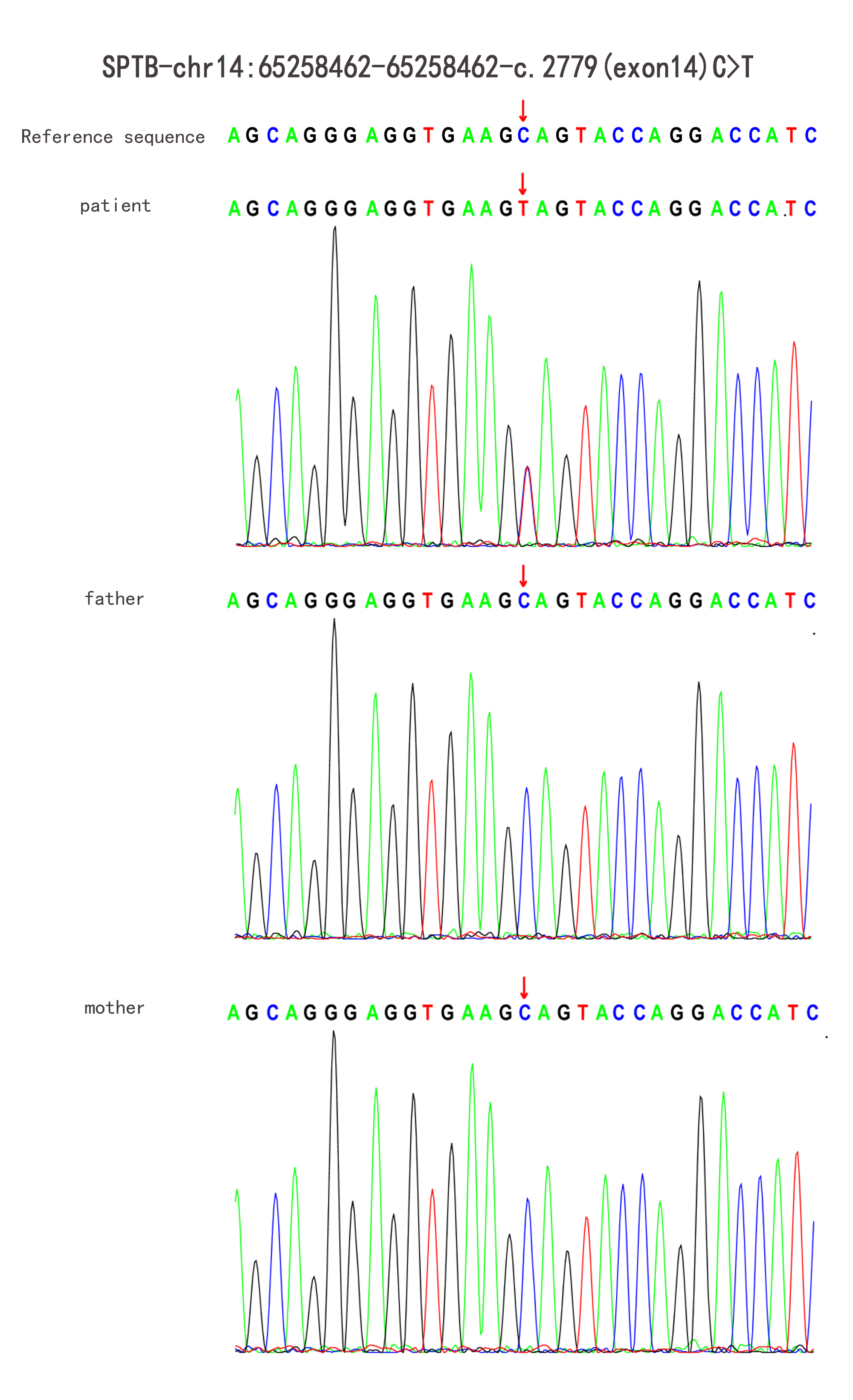

Supplement: Supplementary file 4 [file Presentation_4.zip › supplementary material 4/ID-13.jpg]

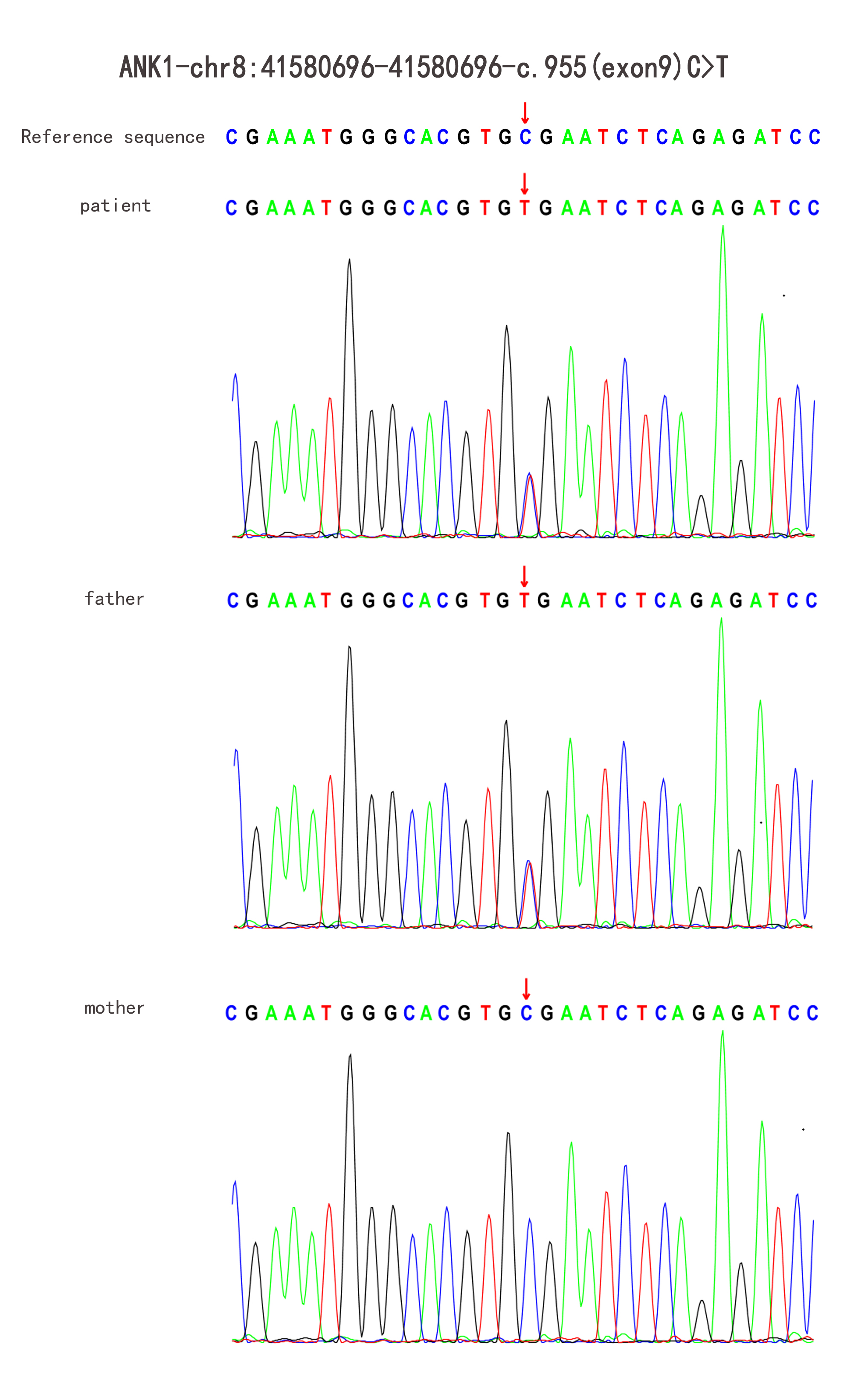

Supplement: Supplementary file 4 [file Presentation_4.zip › supplementary material 4/ID-14.jpg]

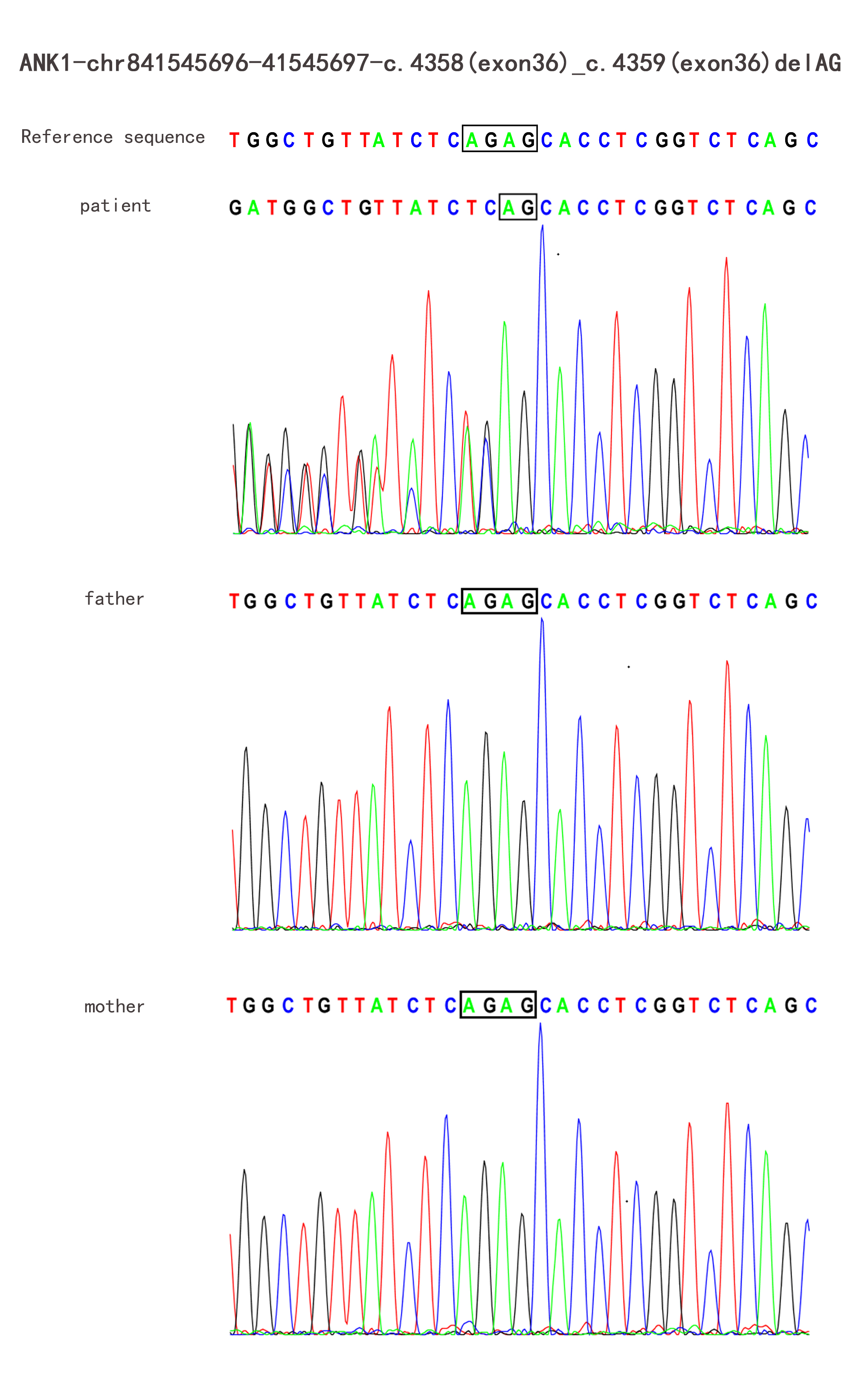

Supplement: Supplementary file 4 [file Presentation_4.zip › supplementary material 4/ID-2.jpg]

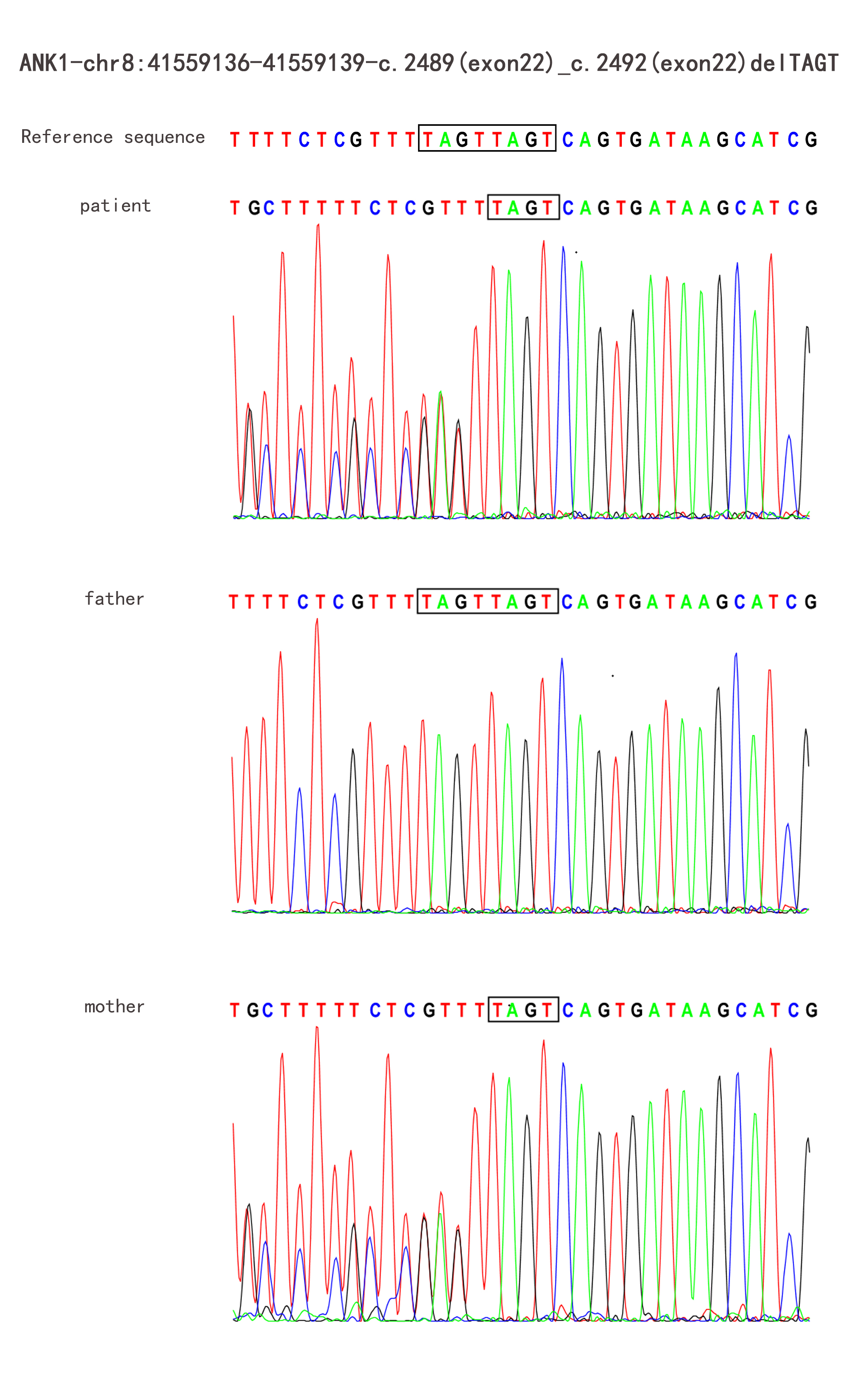

Supplement: Supplementary file 4 [file Presentation_4.zip › supplementary material 4/ID-3.jpg]

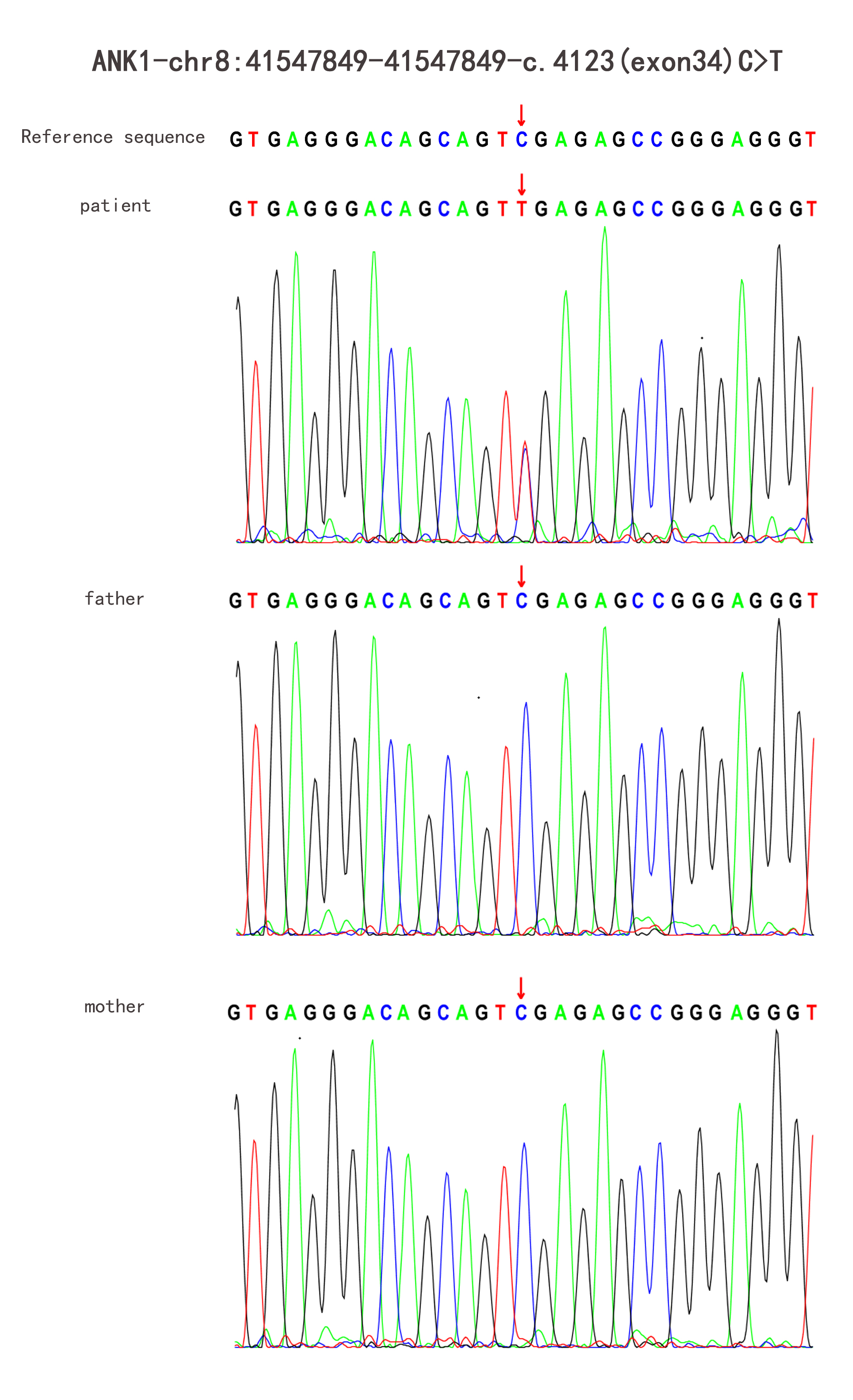

Supplement: Supplementary file 4 [file Presentation_4.zip › supplementary material 4/ID-4.jpg]

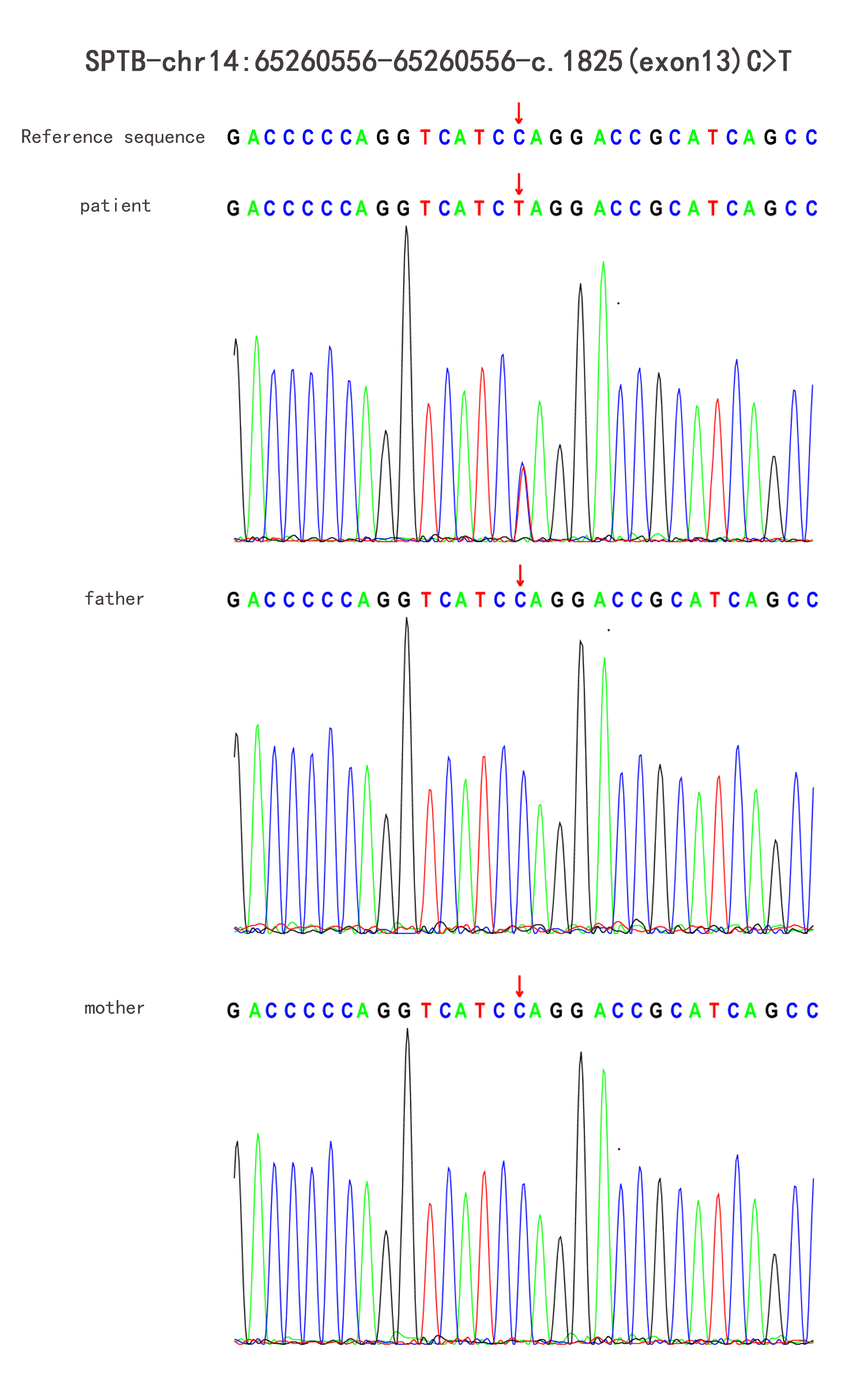

Supplement: Supplementary file 4 [file Presentation_4.zip › supplementary material 4/ID-5.jpg]

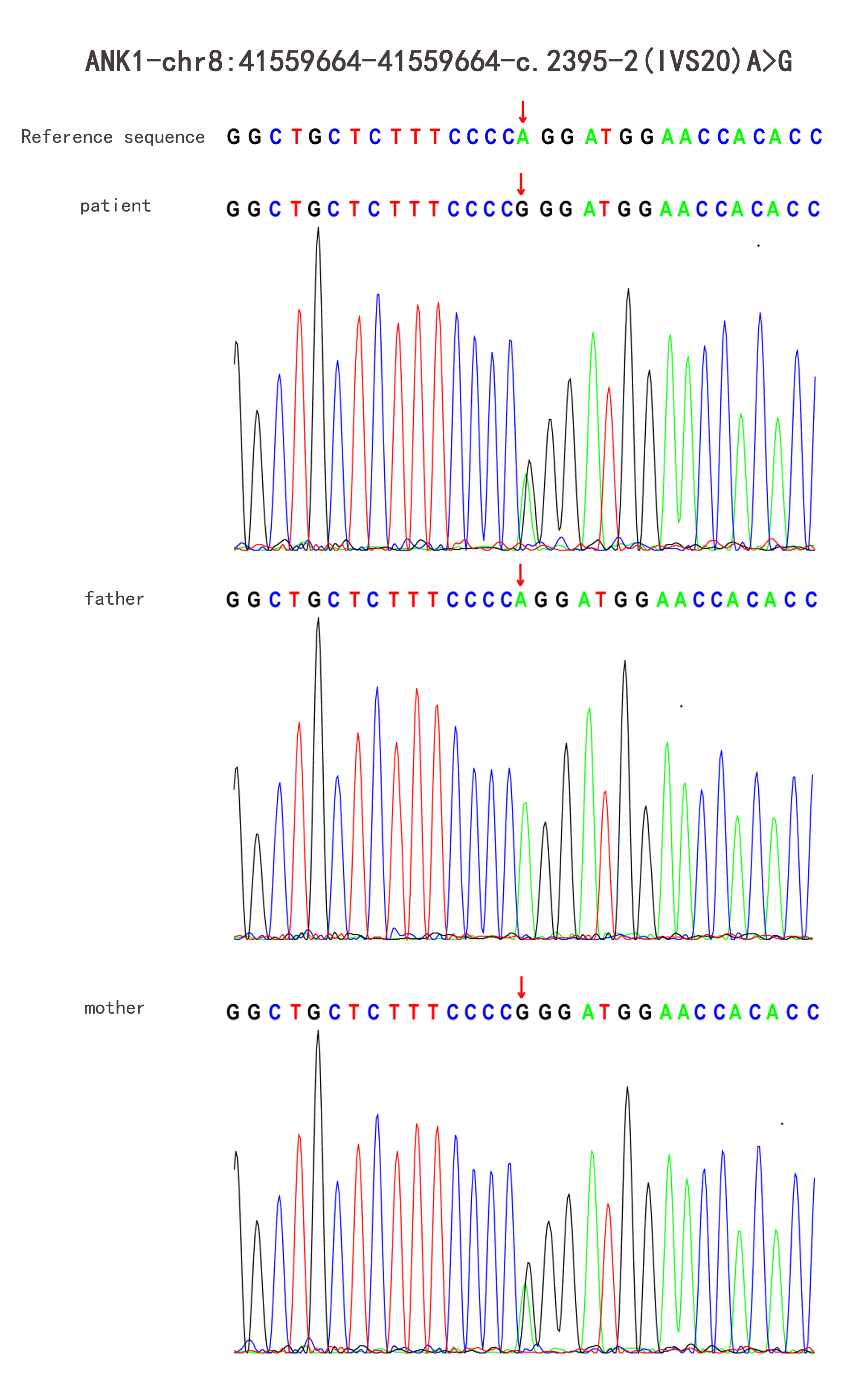

Supplement: Supplementary file 4 [file Presentation_4.zip › supplementary material 4/ID-6.jpg]

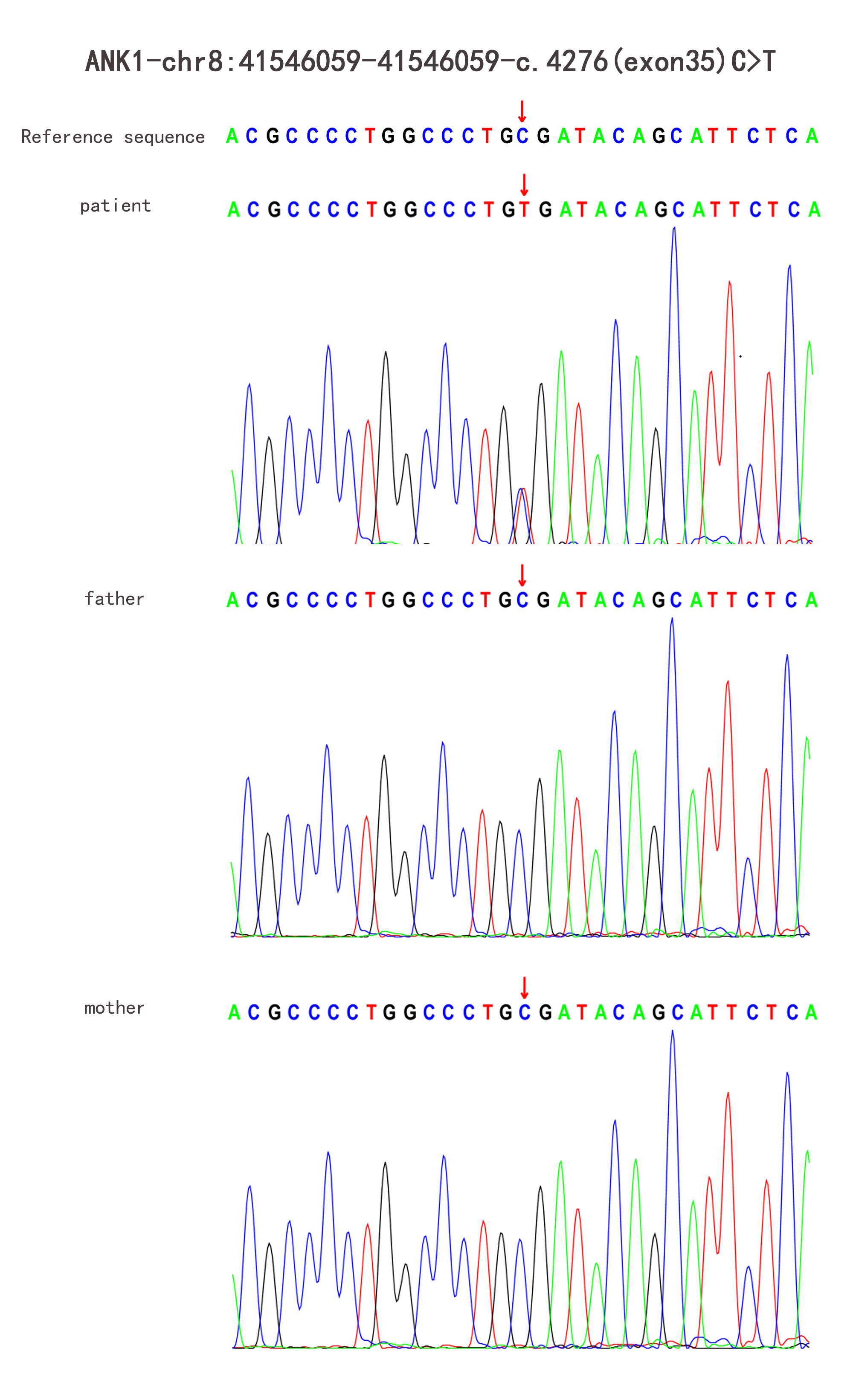

Supplement: Supplementary file 4 [file Presentation_4.zip › supplementary material 4/ID-7.jpg]

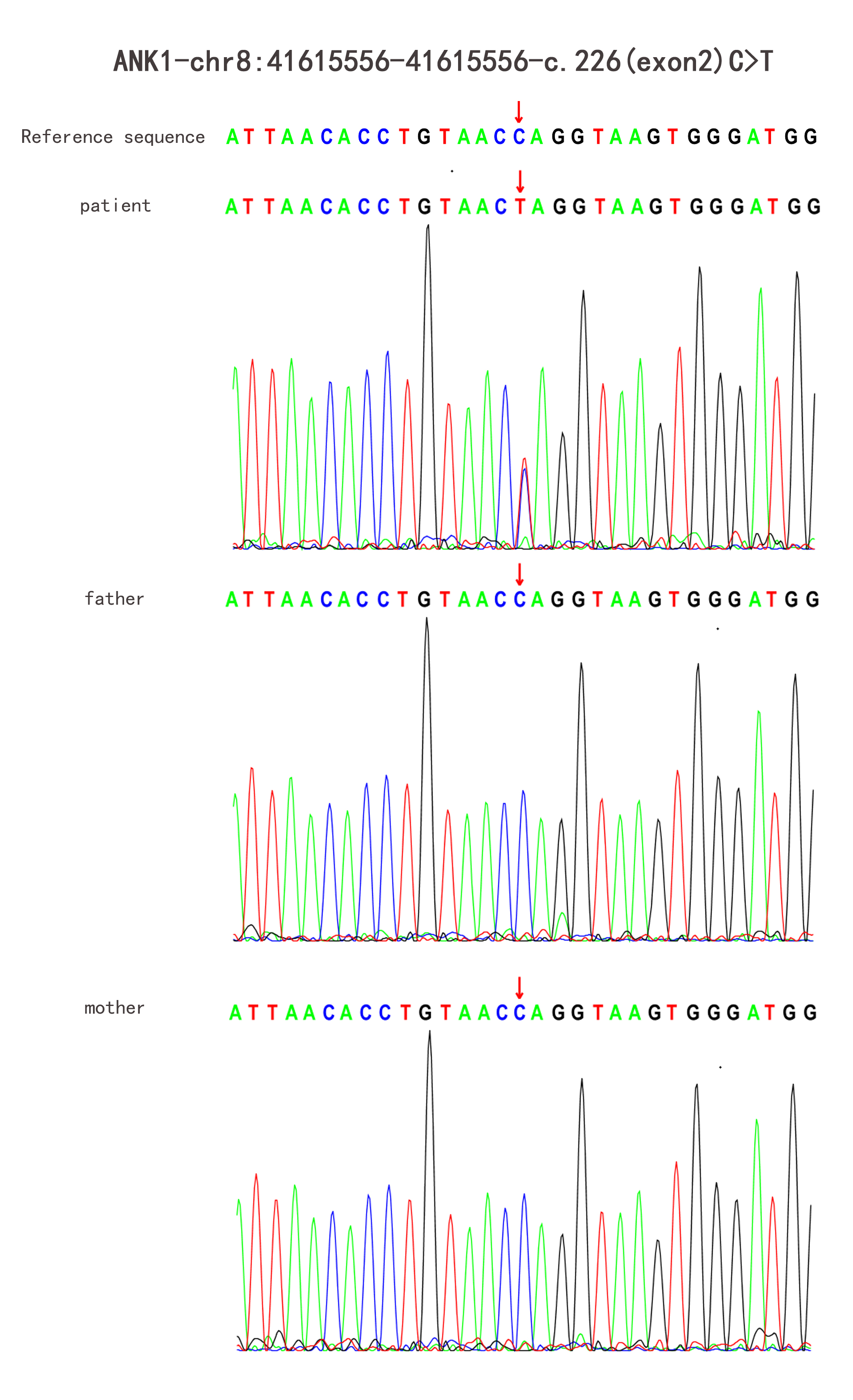

Supplement: Supplementary file 4 [file Presentation_4.zip › supplementary material 4/ID-8.jpg]

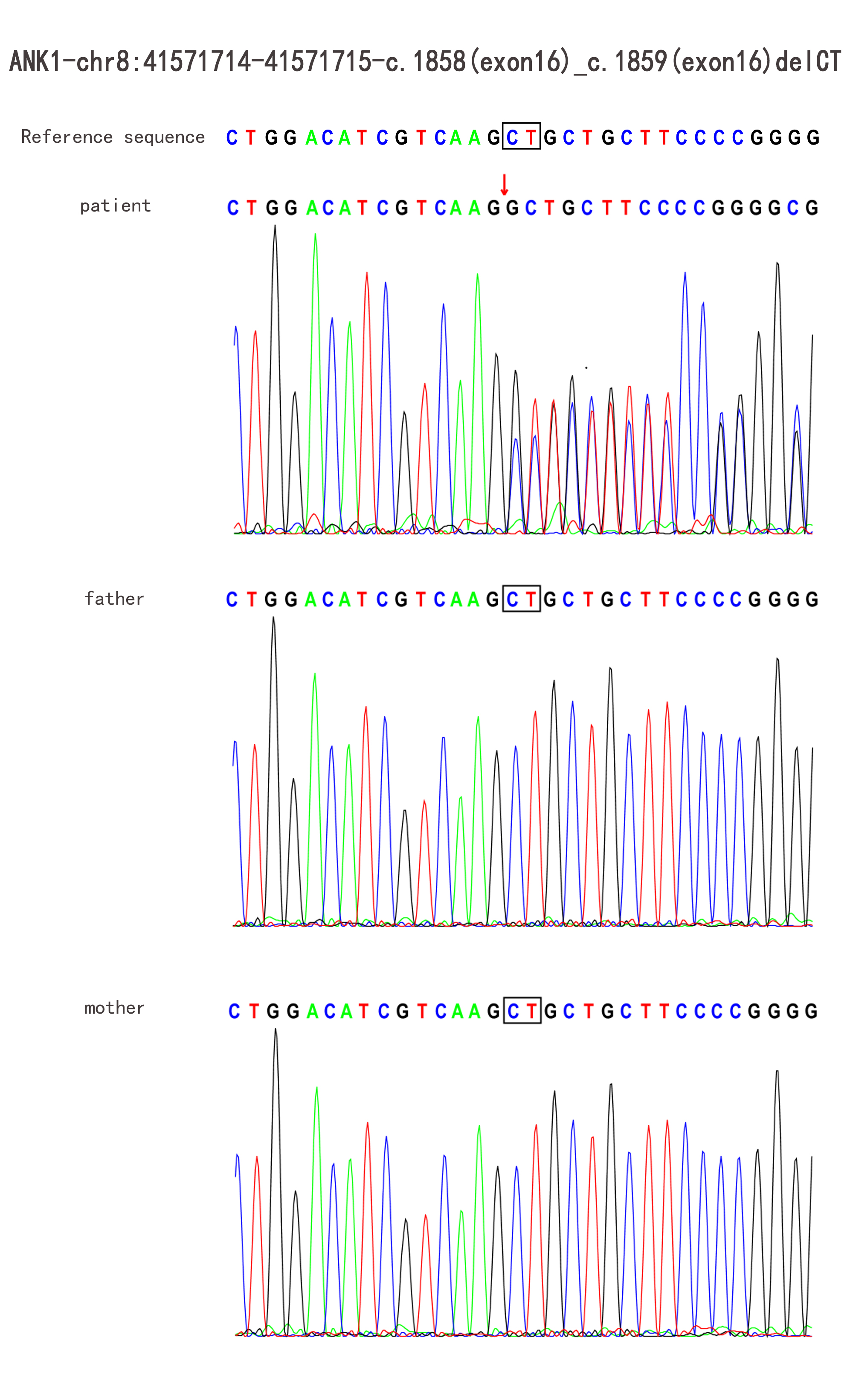

Supplement: Supplementary file 4 [file Presentation_4.zip › supplementary material 4/ID-9.jpg]
