# Supplementary material for: Preliminary Study on the Clinical and Genetic Characteristics of Hereditary Spherocytosis in 15 Chinese Children
Source: Front Genet. 2021 Mar 18;12:652376. doi: 10.3389/fgene.2021.652376 (PMC8044778; doi:10.3389/fgene.2021.652376)
Supplement: Supplementary file 5 [file Presentation_5.pdf]

The protein structure of the mutation site SPTB:NM_001024858: Exon23: C. 4860T>C was predicted by I-Tasser and Pymol software and the model was constructed. 12 bioinformatics software, such as SIFT and Polyphen2, were used to predict the harmousness of variation. Species conservatism of the loci was analyzed by UCSC database.

1. Results of protein structure prediction model construction

After analysis, SPTB:NM_001024858: Exon23: C. 4860T>C is a synonym mutation (Figure 1), which encodes isoleucine at position 1620 before and after mutation and does not cause changes in amino acids. However, the comparison of the structure prediction of the mutant protein is based on the change of amino acids. Therefore, there is no protein structure prediction result for this mutation.

HGVS-compliant variant descriptions

Type	Variant Description	Link to Reference sequence Record
Transcript (:c.)	NM_001024858.2:c.4860T>C	NM_001024858.2
RefSeq Gene (:g.)	NG_016202.1:g.53639T>C	NG_016202.1
Protein (:p.)	NP_001020029.1:p.(Ile1620=)	NP_001020029.1
Protein (:p.)	NP_001020029.1:p.(I1620=)	NP_001020029.1

Genomic Variants

Variant Description	VCF Description	Link to GenBank
NC_000014.9:g.64774510A>G	GRCh38:14:64774510:A:G	NC_000014.9
NC_000014.8:g.65241228A>G	GRCh37:14:65241228:A:G	NC_000014.8

Figure 1. Screenshot of HGVS standard naming

2.Prediction results of 12 bioinformatics software on mutation harmfulness：Twelve bioinformatics software, such as SIFT and Polyphen2, could not predict the harmfuty of the mutation.

Mutation 核酸改变	SPTB:NM_001024858:exon23:c.4860T>C
SIFT_score	/
SIFT_pred	/
Polyphen2_HDIV_score	/
Polyphen2_HDIV_pred	/
Polyphen2_HVAR_score	/
Polyphen2_HVAR_pred	/
LRT_score	/
LRT_pred	/
MutationTaster_score	/
MutationTaster_pred	/
MutationAssessor_score	/
MutationAssessor_pred	/
FATHMM_score	/
FATHMM_pred	/

**PROVEAN_score**	/
**PROVEAN_pred**	/
**MetaSVM_score**	/
**MetaSVM_pred**	/
**MetaLR_score**	/
**MetaLR_pred**	/
**M-CAP_score**	/
**M-CAP_pred**	/
**fathmm-MKL_coding_score**	/
**fathmm-MKL_coding_pred**	/

Note:"/" indicates that the database has no comment information for this locus; SIFT_PRED software corresponding website: http://sift.jcvi.org; Polyphen2_HDIV_pred software corresponding to the website: http://genetics.bwh.harvard.edu/pph2; Polyphen2_HVAR_pred software corresponding to the website: http://genetics.bwh.harvard.edu/pph2; LRT_pred software corresponding to the website: http://www.genetics.wustl.edu/jflab/lrt_query.html; MutationTaster_pred software corresponding to the website: http://www.mutationtaster.org/; MutationAssessor_pred software corresponding to the website: http://mutationassessor.org/r3/; FATHMM_pred software corresponding to the website: http://fathmm.biocompute.org.uk; PROVEAN_pred software corresponding to the website: http://provean.jcvi.org/index.php; MetaSVM_pred software corresponding to the website: https://omictools.com/meta-svm-tool; MetaLR_pred software corresponding to the website: http://www.ensembl.info/tag/metalr/; CAP_pred software corresponding to the website: http://bejerano.stanford.edu/mcap/; Fathmm MKL_coding_pred software corresponding website: http://fathmm.biocompute.org.uk/fathmmMKL.htm.
Rating reference: https://brb.nci.nih.gov/seqtools/colexpanno.html#dbnsfp.

2. Species conservatism analysis results of UCSC loci

Mutation information: SPTB:NM_001024858: Exon23: C. 4860T>C

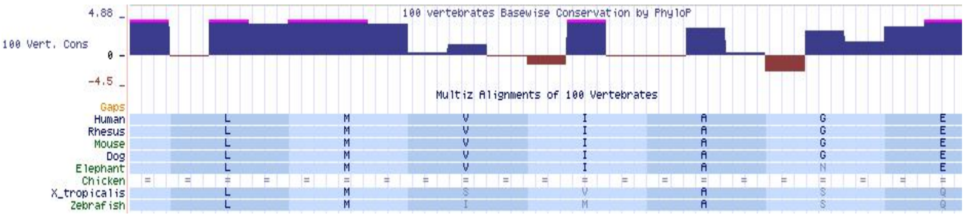

Fig. 2. Species conservatism at loci

As shown in the figure, this locus encodes consistent amino acids in Human, Rhesus, Mouse, Dog, and Elephant. But the amino acids encoded in X_tropicalis, Zebrafish, are different.
