# Supplementary material for: Preliminary Study on the Clinical and Genetic Characteristics of Hereditary Spherocytosis in 15 Chinese Children
Source: Front Genet. 2021 Mar 18;12:652376. doi: 10.3389/fgene.2021.652376 (PMC8044778; doi:10.3389/fgene.2021.652376)
Supplement: Supplementary file 6 [file Presentation_6.pdf]

ID	ACMG	Conservation		
		GERP	phyloP	phastCons
1	Pathogenic	D(3.28)	D(1.166000)	D(0.782000)
2	Pathogenic	-	-	-
3	Pathogenic	-	-	-
4	Pathogenic	D(5.08)	D(1.176000)	D(0.950000)
5	Pathogenic	D(3.01)	D(1.176000)	D(1.000000)
6	Pathogenic	D(4.8)	D(1.138000)	D(0.887000)
7	Pathogenic	D(0.146)	N(-0.293000)	D(0.943000)
8	Pathogenic	D(5.54)	D(1.176000)	D(1.000000)
9	Pathogenic	-	-	-
10	Pathogenic	D(4.89)	D(1.018000)	D(0.971000)
11	Likely pathogenic	N(-3.46)	N(-0.397000)	D(0.891000)
12	Pathogenic	D(0.146)	N(-0.437000)	D(0.775000)
13	Pathogenic	N(-5.29)	D(0.953000)	D(0.994000)
14	Pathogenic	D(2.49)	D(0.017000)	D(0.999000)
15	Uncertain significance	-	-	-
